# Chitosan/Carboxymethyl Cellulose Nanocomposites Prepared via Electrolyte Gelation–Spray Drying for Controlled Ampicillin Delivery and Enhanced Antibacterial Activity

**DOI:** 10.3390/polym18030319

**Published:** 2026-01-24

**Authors:** Anh Dzung Nguyen, Vinh Nghi Nguyen, Vu Hoa Tran, Huu Hung Dinh, Dinh Sy Nguyen, Thi Huyen Nguyen, Van Bon Nguyen, San Lang Wang

**Affiliations:** 1 Institute of Biotechnology and Environment, Tay Nguyen University, Buon Ma Thuot 630000, Vietnam; vuhoa.lab@gmail.com (V.H.T.); ndsy@ttn.edu.vn (D.S.N.); sinhvienk08@gmail.com (T.H.N.); nvbon@ttn.edu.vn (V.B.N.); 2 Ninh Thuan Hospital, Khanh Hoa 650000, Vietnam; nguyenvinhnghi0607@gmail.com; 3 Faculty of Medicine and Pharmacy, Tay Nguyen University, Buon Ma Thuot 630000, Vietnam; dhhung@ttn.edu.vn; 4 Department of Chemistry, Tamkang University, New Taipei 25137, Taiwan

**Keywords:** chitosan, carboxymethyl cellulose, nanocomposite, ampicillin, spray drying, antibacterial activity

## Abstract

This study reports the fabrication of chitosan/carboxymethyl cellulose (C/M) nanocomposites by electrolyte gelation–spray drying and the evaluation of their antibacterial performance as carriers for the antibiotic ampicillin. Chitosan (C), a cationic biopolymer derived from chitin, was combined with the anionic polysaccharide carboxymethyl cellulose (M) at different mass ratios to form stable nanocomposites via electrostatic interactions and then collected in a spray dryer. The resulting particles exhibited mean diameters ranging from 800 to 1500 nm and zeta potentials varying from +90 to −40 mV, depending on the C/M ratio. The optimal formulation (C/M = 2:1 ratio) achieved a high recovery yield (71.1%), lower PDI (0.52), and ampicillin encapsulation efficiency EE (82.4%). Fourier transform infrared spectroscopy (FTIR) confirmed the presence of hydrogen bonding and ionic interactions among C/M, and ampicillin within the nanocomposite matrix. The nanocomposites demonstrated controlled ampicillin release and pronounced antibacterial activity against *Staphylococcus aureus*, with minimum inhibitory concentration (MIC) and minimum bactericidal concentration (MBC) values of 3.2 µg/mL and 5.3 µg/mL, respectively, which were lower than those of free ampicillin. These results indicate that the chitosan/carboxymethyl cellulose nanocomposites are promising, eco-friendly carriers for antibiotic delivery and antibacterial applications.

## 1. Introduction

Chitosan, a derivative of chitin, is a linear natural polymer widely distributed in the shells of crustaceans, insects, and fungal cell walls. Due to its natural origin, biocompatibility, biodegradability, and low toxicity, chitosan has been extensively investigated as a promising material for drug delivery systems. As a naturally occurring polycation (poly-NH_4_^+^), chitosan readily interacts with negatively charged biological membranes and biomolecules, facilitating drug encapsulation and cellular uptake [1,2].

At the nano scale, nanochitosan offers several advantages for drug delivery applications, including high surface area, tunable surface charge, and improved interaction with biological barriers. In addition to its intrinsic antibacterial activity, nanochitosan can enhance drug bioavailability by improving drug stability, prolonging residence time, and enabling controlled or sustained drug release. The interaction between positively charged nanochitosan and negatively charged bacterial membranes or nucleic acids further contributes to the synergistic antibacterial efficacy of drug-loaded systems [3,4].

Nanochitosan has been widely employed as a carrier for various antibiotics, such as ampicillin, amoxicillin, ciprofloxacin, chlortetracycline, and gentamicin, with the aim of enhancing antibacterial efficacy and overcoming antibiotic resistance [5,6,7,8]. Nevertheless, several limitations hinder its practical application in drug delivery, including high production costs, insufficient particle stability under acidic conditions, and relatively low drug loading and release efficiency associated with the linear molecular structure of chitosan. Moreover, many conventional nanochitosan fabrication methods—except spray drying—suffer from low recovery yields and the use of undesirable chemical reagents, which restrict their scalability and biomedical applicability [9].

Cellulose, similar to chitosan, are natural, biocompatible, and biodegradable polymers that have also been explored as drug carriers in the form of nanoparticles, nanofilms, and nanofibers. However, drug delivery systems based solely on starch or cellulose often exhibit poor mechanical properties, limited structural stability, and rapid enzymatic degradation, leading to uncontrolled drug release and reduced therapeutic efficiency [10,11]. Therefore, to improve drug loading capacity, structural stability, and release performance while reducing production costs, chitosan can be combined with negatively charged polymers such as alginate, cellulose, and starch to form nanocomposite or multilayered drug delivery systems via electrostatic interactions. These nanocomposites provide enhanced network structures, improved physicochemical stability, and greater control over drug encapsulation and release, making them highly promising platforms for advanced drug delivery applications [12,13,14,15].

Nevertheless, chitosan-based systems still suffer from inherent limitations, such as poor structural stability, low drug loading efficiency, and insufficient control over drug release, which are primarily attributed to the linear molecular architecture of chitosan. In addition, many reported fabrication methods involve complex processes, low recovery yields, or the use of chemical crosslinkers, which restrict scalability and biomedical applicability. Comparative investigations on the effects of polymer composition and molecular weight on drug delivery performance, particularly for nanocomposite systems produced by scalable techniques such as spray drying, are still limited. Therefore, this study aims to develop chitosan-based nanocomposite drug delivery systems by combining chitosan with carboxymethyl cellulose to improve drug loading efficiency, structural stability, and controlled release behavior. The findings are expected to contribute to the rational design of efficient and cost-effective polysaccharide-based nanocarriers for drug delivery applications.

## 2. Materials and Methods

### 2.1. Materials

Chitosan (Sigma, St. Louis, MO, USA; Mw ≈ 500 kDa, degree of deacetylation > 75%, purity >95%) was used as the primary polymer. Sodium carboxymethyl cellulose (CMC) was obtained from Merck (Darmstadt, Germany). Ampicillin sulfate was purchased from Sigma-Aldrich (St. Louis, MO, USA). Nutrient Broth was supplied by Himedia (Maharashtra, India). *Staphylococcus aureus* ATCC 25923 was provided by the Pasteur Institute, Ho Chi Minh City, Vietnam.

### 2.2. Preparation of Ampicillin-Loaded Chitosan/CMC Nanocomposites

Chitosan (0.1% *w*/*v*) was dissolved in 0.1 N acetic acid and adjusted to pH 5.0 using 0.01 N NaOH. CMC (0.1% *w*/*v*) was prepared separately in deionized water (pH 7.0). Ampicillin was incorporated into the chitosan/CMC mixtures to achieve final drug concentrations ranging from 0 to 7.5 µg/mL. Chitosan (C) and CMC (M) solutions were combined at different weight ratios and magnetically stirred for 60 min at room temperature. The final pH of the nanocomposite suspensions was 5.2.

Nanocomposite particles were spontaneously formed via electrolyte gelation between positively charged chitosan and negatively charged CMC. The suspensions were spray-dried using a nano spray dryer (B-90, Büchi, Flawil, Switzerland) under optimized conditions: inlet temperature 120 °C, outlet temperature 80 °C, feed rate 120 mL/h, compressed air flow 1.2 m^3^/min, and nozzle diameter 5.5 µm [16]. All formulations were prepared in triplicate.

### 2.3. Characterization of Nanocomposites

Nanoparticle morphology was characterized by scanning electron microscopy (SEM, Phenom ProX, Thermo Scientific, Waltham, MA, USA). The mean particle size, size distribution, and zeta potential were determined using a Nanosizer SZ-100 (Horiba, Kyoto, Japan) at 25 °C in PBS (pH 7.0). Measurements were performed in triplicate using a dispersion medium viscosity of 0.895 mPa·s. The electrode voltage was set to 3.3 V (zeta mode), and the sample concentration was adjusted to 1 mg/mL.

Chemical interactions between chitosan, ampicillin, and CMC were analyzed by FT-IR spectroscopy (Alpha, Bruker, Billerica, MA, USA) in the range of 500–4000 cm^−1^.

### 2.4. Ampicillin Encapsulation Efficiency and Release Kinetics

The encapsulation efficiency (EE %) and release behavior of ampicillin were determined using a centrifugation-based method adapted from Schumacher (1997) [17]. Briefly, 50 µg of nanocomposite was dispersed in PBS (pH 7.0) and centrifuged at 14,000× *g* rpm at 5 °C. The supernatant containing the un-encapsulated drug was isolated, and the nanocomposite containing encapsulated ampicillin was resuspended in drug-free PBS buffer (pH = 7.0) for the test of release kinetics at room temperature for 24 h.

Ampicillin concentration was quantified by HPLC (UHPLC Thermo 3000) equipped with a Hypersil GOLD PFP column using MeOH/ACN/HCOOH (30:40:30, *v*/*v*/*v*) as the mobile phase at a flow rate of 0.2 mL/min and detection wavelength of 270 nm. Encapsulation efficiency was calculated as:

EE (%) = [(Qt − Qs)/Qt] × 100

where *Qₜ* is the total initial amount of ampicillin and *Qₛ* is the amount of non-encapsulated ampicillin.

### 2.5. Antibacterial Activity Assay

Antibacterial activity was evaluated against *S. aureus* (10^6^ CFU/mL) cultured in Nutrient Broth containing ampicillin-loaded chitosan/CMC nanocomposites (0–7.5 µg/mL). Cultures were incubated at 37 °C for 48 h with shaking at 120 rpm. Bacterial growth was monitored by measuring optical density at 620 nm using a UV-Vis spectrophotometer (Jasco, Hachioji, Japan). Antibacterial efficacy was expressed as inhibition (%) [18].

Inhibition (%) = [(OD_control_ − OD_sample_)/OD_control_] × 100

in which OD_control_ is an average OD620 index of the control, and OD_sample_ is an average OD620 index of samples that encapsulated ampicillin.

### 2.6. Determination of MIC and MBC

Minimum inhibitory concentration (MIC) was determined using the broth dilution–resazurin method, while minimum bactericidal concentration (MBC) was assessed by agar diffusion. MIC and MBC values were defined as the lowest concentrations preventing visible bacterial growth after incubation at 37 °C for 24 h [19]. In brief, a volume of 1.0 mL of the culture exposed to the nanocomposites that encapsulated ampicillin was inoculated in 9.0 mL of NaCl (0.9% *w*/*v*), and serial dilutions were made up to 10^−5^. The resulting bacterial suspension (1000 µL) was spread and plated onto a Nutrient Broth Agar plate and incubated at 37 °C for 24 h. After incubation, the CFUs that appeared on the Nutrient Broth Agar plate were counted, where the result was expressed as CFU/mL. The lowest concentration that killed 99.9% of the starting inoculum was defined as the MBC.

### 2.7. Statistical Analysis

All experiments were performed in triplicate. Data were analyzed using one-way ANOVA followed by Duncan’s multiple range test (*p* < 0.05) with SPSS 20.0. Results are presented as mean ± standard deviation (SD).

## 3. Results and Discussion

### 3.1. Effect of Chitosan/Carboxymethyl Cellulose Ratio on the Properties of Chitosan/Cellulose Nanocomposites Prepared by Electrolyte Gelation–Spray Drying

The interplay between chitosan and carboxymethyl cellulose (CMC) in polyelectrolyte complexes critically determines the physicochemical properties of the resulting composite particles. Chitosan, a cationic polysaccharide, interacts electrostatically with the anionic carboxylate groups of CMC, leading to complexation that strongly influences particle size, surface charge, and stability upon spray drying. Such ionic interactions have been increasingly investigated for tailored nanocomposite materials with enhanced functional properties [20].

#### 3.1.1. Particle Size and Morphology

**Figure 1:**
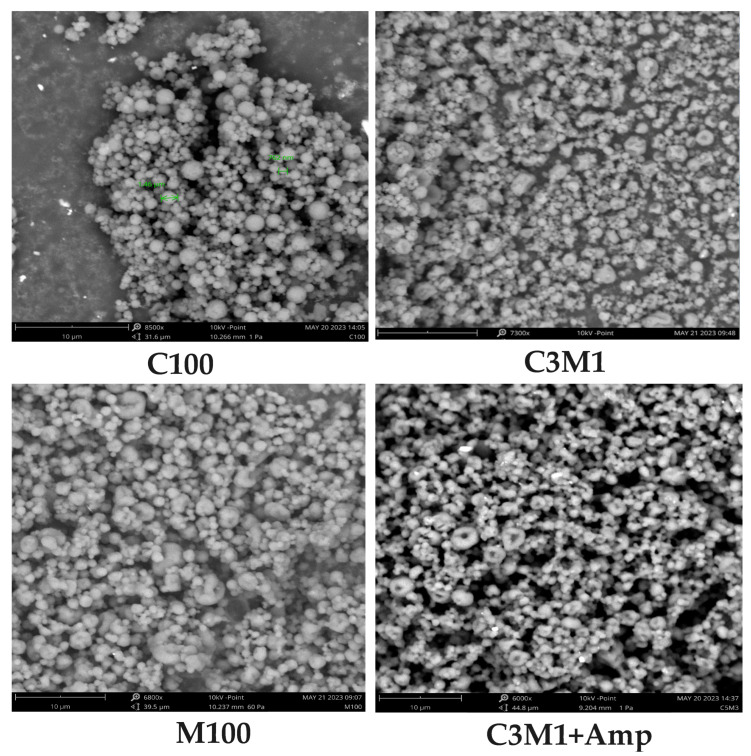
SEM images of chitosan/carboxymethyl cellulose nanocomposite and nano chitosan/carboxymethyl cellulose composite loaded ampicillin. (Taken by SEM, Phenome ProX, Thermo, Waltham, MA, USA; 8000×, 10 kV, bar 10 µm). C100: Data of this sample is from the paper [21].

As summarized in Table 1, Figure 1 the mean particle diameter of chitosan/CMC nanocomposites varied systematically with composition. Pure chitosan (C100) formed relatively small particles (~816 nm), whereas increasing CMC content led to progressively larger particles—reaching approximately 1900 nm at the highest CMC ratio (C1M5). This trend reflects the increased prevalence of ionic crosslinks and the concomitant formation of dense polyelectrolyte complexes that aggregate into larger clusters prior to spray drying. The gradual increase in particle size with higher CMC content suggests stronger electrostatic interactions and complexation kinetics that favor the formation of larger precursors before atomization and solvent removal. These observations align with recent work demonstrating the importance of polyelectrolyte balance on particle formation mechanisms in spray-dried systems [12,20].

In contrast to our findings with CMC, previous studies on chitosan-based composites have reported different particle size trends depending on the complementary polymer and processing method. While particle size in our chitosan/CMC system increased with higher CMC content, other studies showed that increasing polysaccharides such as starch led to reduced particle sizes. This difference highlights the crucial role of polymer charge density and molecular architecture in controlling complexation behavior and spray drying outcomes [21,22]. These contrasting trends arise mainly from differences in nanocomposite formation mechanisms. In this study, chitosan/CMC nanoparticles were formed in solution via electrolyte gelation prior to spray drying, meaning particle size was established before drying and depended on the polymer ratio, and stirring conditions. In contrast, chitosan/starch systems prepared by direct spray drying do not form nanoparticles in solution beforehand, resulting in fundamentally different particle formation mechanisms [21,22].

#### 3.1.2. Zeta Potential and Colloidal Stability

**Figure 2:**
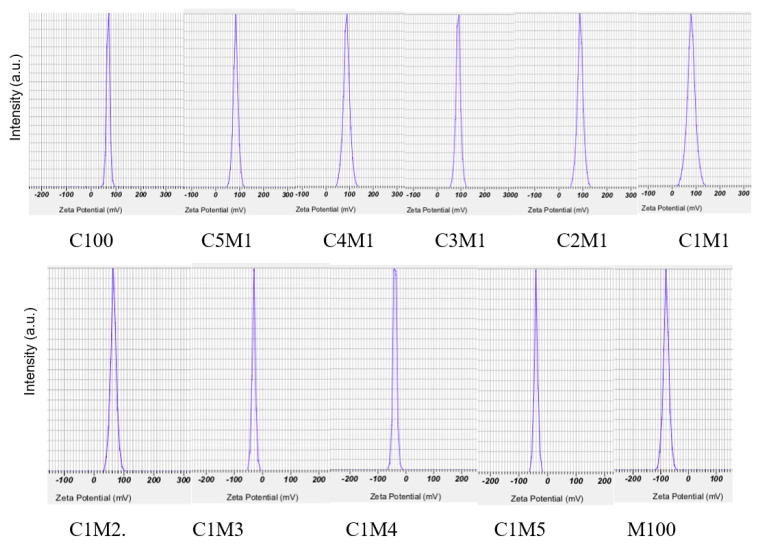
Effect of chitosan/carboxymethyl cellulose ratio on the zeta potential of the nanocomposite (analyzed by a Nanosizer SZ 100, Horiba, Kyoto, Japan, at 25 °C) (Zeta Supplement). C100: Data of this sample is from the paper [21]. (Figure S1 in Supplement Materials).

Zeta potential measurements further elucidated the impact of the polymer ratio on surface charge. The size distribution of all samples ranged from 100 to 1900 nm. As shown in Table 1 and Figure 2 decreasing the chitosan fraction systematically reduced the net positive surface charge due to the increasing contribution of negatively charged CMC. High absolute zeta potentials (>|30| mV) are generally associated with improved colloidal stability by electrostatic repulsion, which is consistent with the strong charge interactions observed in our chitosan/CMC complexes.

The recent literature on spray-dried chitosan microparticles corroborates the critical role of charge balance and processing conditions on zeta potential and stability. Specifically, reviews of inhalable chitosan-based spray-dried composites report that both formulation parameters and drying techniques significantly influence charge distribution, particle aggregation, and aerodynamic performance of chitosan-containing particles [16,20].

More broadly, recent advances in polyelectrolyte-complexed biopolymer particles continue to highlight how subtle differences in polymer charge density, molecular weight, and processing parameters can dramatically alter morphology, surface charge, and functionality. For example, chitosan/CMC systems with incorporated functional nanoparticles (e.g., silver) have been shown to yield enhanced antimicrobial and hemostatic properties, further illustrating how tailored composition can tune both physical and biological performance [12].

Understanding the relationship between the polymer ratio, complexation behavior, and the resulting physicochemical properties is essential for designing functional nanocomposites for applications such as drug delivery, hemostatic materials, and environmentally responsive carriers. The ability to tune particle size and surface charge via simple formulation adjustments provides a versatile platform for exploiting chitosan/CMC interactions in diverse contexts.

The antibacterial activity of chitosan is closely related to its positive surface charge, as reflected by the zeta potential. As shown in Table 1, spray-dried chitosan nanoparticles (C100) obtained in this study exhibited a high zeta potential of +67.67 mV, which is markedly higher than values reported in previous studies (+30 to +55 mV) for chitosan nanoparticles prepared by spray drying or ionic gelation–spray drying combinations [16,20,22,23].

Earlier reports showed that chitosan nanoparticles prepared using sodium tripolyphosphate (TPP) typically possess much lower zeta potentials (+25 to +29 mV), while crosslinked and spray-dried chitosan nanoparticles exhibited values below +35 to +52 mV. These comparisons indicate that the fabrication strategy employed in the present work enables a generation of chitosan nanoparticles with an unusually high surface charge [16,20,22,23].

For chitosan/cellulose nanocomposites, the zeta potential increased significantly with changes in the chitosan-to-CMC ratio, reaching +78.63 mV (C1M1), +85.07 mV (C4M1), +89.53 mV (C3M1), and a maximum of +90.37 mV (C2M1). This enhancement is attributed to strong ionic interactions between the positively charged chitosan and negatively charged CMC, promoting the formation of electrostatically stabilized nanocomposite particles. Based on the zeta potential (Table 1) and FT-IR data (Figure 4), we propose that the nanocomposites exhibit a proposed model as a core–shell-like structure, in which CMC-rich domains form the particle core, while chitosan chains are preferentially enriched at the particle surface, leading to a high density of positive charges. This structural arrangement is supported by FT-IR analysis (Figure 4), which evidences ionic interactions between chitosan and CMC, and is consistent with the higher zeta potential of the chitosan/carboxymethyl cellulose nanocomposites (Table 1) compared with chitosan (C100) and CMC nanoparticles (M100).

When the CMC content exceeded 50%, the zeta potential decreased sharply to +61.67 mV in the C1M2 formulation and eventually shifted to negative values (−31.43 to −40.00 mV for C1M3–C1M5). This transition is attributed to the intrinsically high negative surface charge of CMC (−81.73 mV), which becomes dominant at elevated CMC proportions, thereby overriding the positive charge of chitosan. A similar trend has been reported for chitosan/starch nanocomposites, where starch with its negative zeta potential (−19 mV) progressively reduces the overall surface charge as its proportion increases [21,24].

These findings are consistent with recent studies showing that polymer charge density and composition strongly influence zeta potential and surface properties. Some works reported a significant reduction in zeta potential upon polymer modification and drug loading, highlighting the sensitivity of surface charge to compositional changes [25,26].

#### 3.1.3. Particle Size Distribution and Recovery Yield

**Figure 3:**
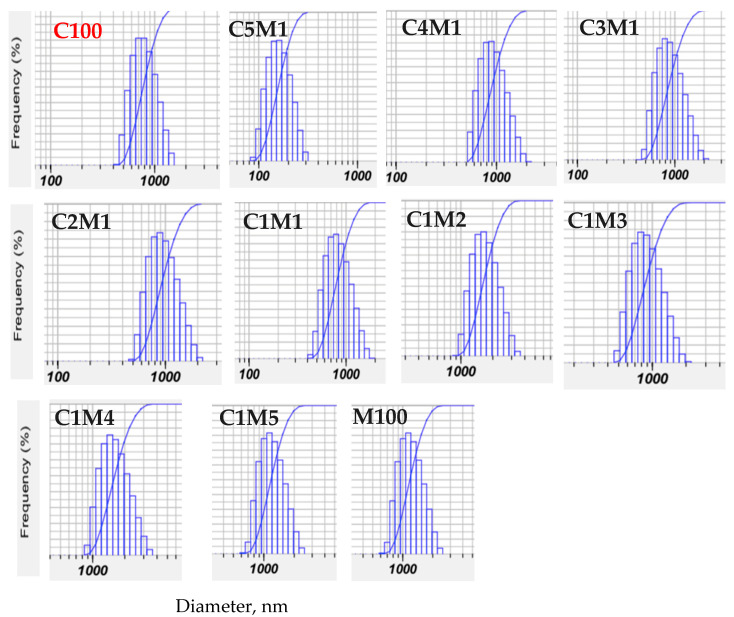
Effect of chitosan/carboxymethyl cellulose ratio on the size distribution of the nanocomposite (analyzed by a Nanosizer SZ 100, Horiba, Kyoto, Japan, at 25 °C). (Size Supplement). C100: Data of this sample is from the paper [21]. (Figure S2 in Supplement Materials).

In nanoparticle systems, PDI (polydispersity index) is a key indicator of particle size uniformity; a lower PDI reflects a narrower and more monodisperse distribution. As shown in Figure 3 and Table 1, the chitosan/carboxymethyl cellulose ratio markedly influences particle size homogeneity. The C3M1 and C2M1 formulations exhibited PDI values of 0.52–0.53, which are considered acceptable for controlled drug release applications, and lower than those of the chitosan nanoparticles prepared by ionic gelation (0.66–0.68) [27]. Notably, compared with earlier reports describing broad size distributions (1–8 μm) and relatively low zeta potentials, the present nanocomposites demonstrate substantially improved surface charge and more controlled particle formation [28].

Spray drying afforded high recovery yields ranging from 67.01% to 75.49%, with composite formulations achieving yields of approximately 70–72%. These values are comparable to chitosan/starch nanocomposites and significantly higher than yields reported in previous studies (47–62%), confirming the efficiency of combining ionic complexation with spray drying for the production of chitosan-based nanocomposites [20,22,23].

#### 3.1.4. FT-IR Analysis of Chitosan/Carboxymethyl Cellulose Nanocomposites

**Figure 4:**
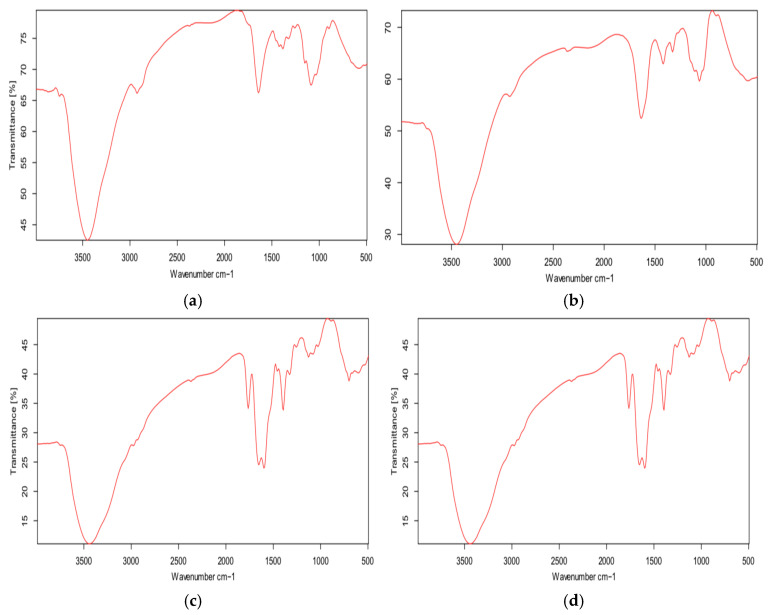
FT-IR of chitosan/carboxymethyl cellulose nanocomposite that encapsulated ampicillin (FT-IR Alpha, Brucker, Billerica, MA, USA with a range of 500–4000 cm^−1^ at a resolution of 16 cm^−1^ within 32 scans). C100 (**a**); M100 (**b**); C2M1 + Amp (**c**); and Amp (**d**). (FT-IR Supplement). C100: Data of this sample is from the paper [21]. (Figure S3 in Supplement Materials).

The FT-IR spectrum of ampicillin (Amp) (Figure 4d) shows characteristic absorption bands at ~3444 cm^−1^, attributed to N–H stretching of amine and amide groups and O–H stretching of carboxylic groups. Bands around 2900 cm^−1^ correspond to C–H stretching vibrations, while the most distinctive peak at 1765 cm^−1^ is assigned to C=O stretching of the β-lactam ring. Additional bands between 1660 and 1599 cm^−1^ are related to N–H bending of primary amine groups [29,30].

The FT-IR spectrum of carboxymethyl cellulose (CMC, M100) (Figure 4b) exhibits typical bands at ~3450 cm^−1^ (O–H stretching), 2927 cm^−1^ (C–H stretching), and 1064 cm^−1^ (C–O–C stretching of glycosidic bonds). The absorption near 1636 cm^−1^ is associated with carboxylate (–COO^−^) groups. Similarly, chitosan (C100) (Figure 4a) displays characteristic bands at ~3449 cm^−1^ (overlapping O–H and –NH_2_ stretching), ~2930 cm^−1^ (C–H stretching), ~1641 cm^−1^ (N–H bending), and ~1064 cm^−1^ (C–O–C stretching) [21,31,32,33].

The FT-IR spectrum of ampicillin-loaded chitosan/cellulose nanocomposites (C2M1 + Amp) (Figure 4c) contains the main characteristic bands of all components, confirming successful incorporation of ampicillin into the composite matrix. Broad absorption around 3450 cm^−1^ and bands near 2900 cm^−1^ arise from overlapping functional groups of Amp, CMC, and chitosan. Importantly, the β-lactam C=O band at 1765 cm^−1^ remains detectable, indicating that the chemical structure of ampicillin is preserved after encapsulation.

Notably, the N–H bending bands of ampicillin at 1660 and 1599 cm^−1^ shift slightly to ~1653 and ~1596 cm^−1^ in the nanocomposite, providing evidence for hydrogen bonding and/or ionic interactions between ampicillin and functional groups of the composite, particularly hydroxyl groups of CMC and protonated amino groups (–NH_3_^+^) of chitosan [33,34].

Overall, the FT-IR results confirm the formation of a network-like chitosan/carboxymethyl cellulose nanocomposite stabilized by ionic interactions and hydrogen bonding. These interactions contribute to structural stability and are expected to retard ampicillin release from the particles. Combined with zeta potential data, the FT-IR analysis supports a proposed model as a core–shell-like structure, in which negatively charged CMC forms the core, while positively charged chitosan is enriched at the particle surface. Ampicillin is preferentially associated with the chitosan-rich outer layer through electrostatic and hydrogen-bonding interactions, explaining the high zeta potential and improved stability observed at optimal chitosan/CMC ratios.

#### 3.1.5. The Release Profile of Ampicillin from Chitosan/Carboxymethyl Cellulose Nanocomposite

**Figure 5:**
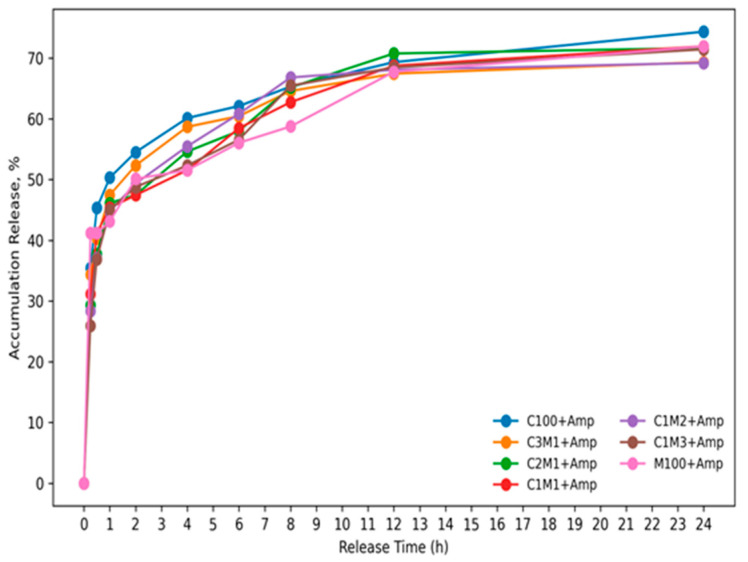
Release profiles of ampicillin from chitosan/carboxymethyl cellulose nanocomposite. Ampicillin concentration of 7.5 μg/mL, in phosphate buffer solution (PBS), pH = 7.0 within 24 h; values in the figure are mean of triplicates ± SE).

The release profiles (Figure 5) show a typical biphasic behavior, consisting of an initial burst release followed by a slower, sustained-release phase. During the early period (≈0–1 h), all formulations exhibit a rapid increase in cumulative drug release, indicating that a fraction of the drug is likely adsorbed/weakly bound at or near the particle surface and is therefore released quickly upon contact with the medium. After this initial stage, the release rate decreases markedly and becomes more gradual from ≈2 h onward, suggesting that drug transport is increasingly governed by diffusion through the polymeric matrix and/or swelling–relaxation of the polymer network (Korsmeyer–Peppas) (R^2^ = 0.92, n = 3), diffusion vs. swelling/relaxation) [21,24].

Notably, the cumulative release approaches a plateau (~70% for the nanocomposite, and 75% for chitosan nanoparticles at 24 h) for most samples, implying that a portion of the drug may remain more strongly entrapped within the matrix or associated with less accessible domains, which is consistent with a controlled, prolonged release system rather than immediate release. Overall, the sustained phase and the late-time plateau support the conclusion that the carrier system provides release retardation, potentially reducing dosing frequency and maintaining therapeutic levels over an extended period.

### 3.2. Antibacterial Activity of Ampicillin-Loaded Chitosan/Cellulose Nanocomposites Against Staphylococcus aureus

*Staphylococcus aureus* is a major opportunistic pathogen commonly colonizing the skin and upper respiratory tract and is responsible for a wide range of infections, particularly in immunocompromised individuals. The rapid emergence of multidrug-resistant *S. aureus*, especially MRSA strains, has significantly reduced the clinical efficacy of conventional β-lactam antibiotics, including ampicillin. Therefore, nano-enabled antibiotic delivery systems that protect antibiotics from enzymatic degradation and enhance antibacterial efficacy represent a promising therapeutic strategy [35].

#### 3.2.1. Effect of Ampicillin Loading on Antibacterial Activity

**Figure 6:**
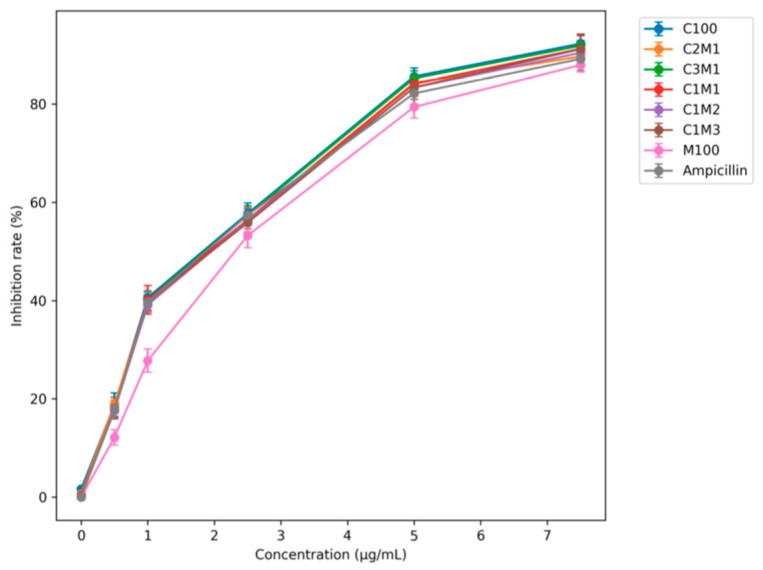
Effect of ampicillin concentration loaded on the nanocomposite on antibacterial activity. *S. aureus* was cultivated in Nutrient Broth, pH = 7.0; at 37 °C for 48 h, and shaking with 120 rpm; values in the figure are mean of triplicates ± SE, *p* < 0.05).

Ampicillin-loaded chitosan/cellulose nano- and microcomposites were prepared with initial ampicillin concentrations ranging from 0 to 7.5 µg/mL prior to spray drying. As summarized in Table 1 and Figure 6, chitosan nanoparticles (C100) exhibited an encapsulation efficiency (EE) of 68.6%, comparable to that of chitosan/starch nanocomposites (60–77%). In contrast, chitosan/cellulose nanocomposites showed significantly higher EE values, reaching 78.8% (C5M1), 81.8% (C3M1), and 82.4% (C2M1), exceeding those reported for chitosan/starch systems (60–77%) [21,24]. Other works reported that cephalosporin antibiotics and β-lactamase inhibitors were loaded into chitosan nanoparticles or ampicillin in chitosan/starch nanocomposites (60–65%). The encapsulation efficiency of cefotaxime and ceftiofur was approximately 70% and 60%, lower than those in this study. Ampicillin was encapsulated in the liposome; however, the reported encapsulation efficiency of ampicillin was 50–60%, which is lower than the efficiency observed in this study [17,21,24,36].

The enhanced encapsulation efficiency is attributed to the formation of a dense ionic network between positively charged chitosan (+67 mV) and highly negatively charged carboxymethyl cellulose (−81 mV). This network retains ampicillin, which carries a net negative charge, within the composite matrix while maintaining a high positive surface charge (+70 to +90 mV). However, further increasing the cellulose fraction resulted in a gradual decrease in EE (75.8% for C1M2, 58.6% for C1M3, and 50.8% for C1M5). Pure CMC nanoparticles (M100) exhibited the lowest EE (≈38%), due to electrostatic repulsion between negatively charged CMC and ampicillin and the absence of a network structure.

Consistent with the encapsulation efficiency, the antibacterial inhibition of M100 + Amp (≈87%) was lower than that of free ampicillin (≈92%) at 7.5 µg/mL. In contrast, C100 + Amp and C2M1 + Amp achieved inhibition rates of approximately 93.5%. Considering the encapsulation efficiency (70–80%), the minimum inhibitory concentration (MIC) of ampicillin-loaded chitosan/CMC nanocomposites against *S. aureus* was reduced to 3.0–4.0 µg/mL, comparable to or lower than values reported for other nano-delivery systems, from around 5.0 to 12.5 [20,21,23,24,37,38].

#### 3.2.2. Time-Dependent Antibacterial Performance and Anti-Resistance Effect

**Figure 7:**
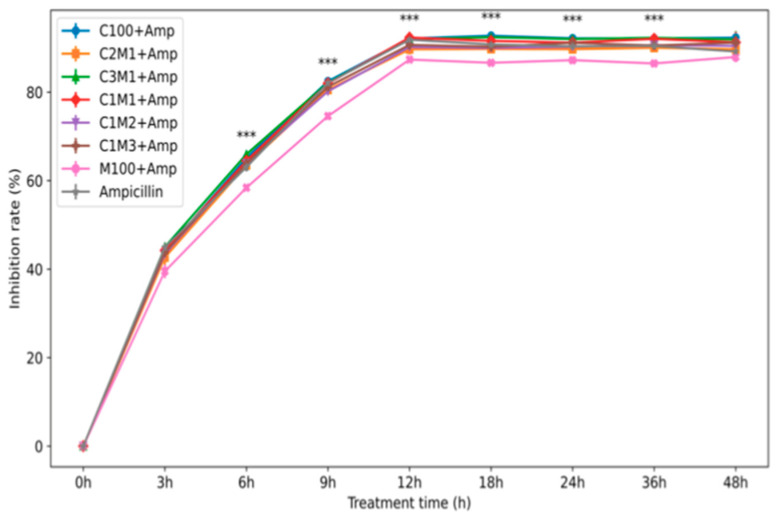
Effectiveness of chitosan/carboxymethyl cellulose loaded ampicillin on *S. aureus* antibacterial activity. *Staphylococcus aureus* was cultivated in Nutrient Broth, pH = 7.0; at 37 °C for 48 h, and shaking with 120 rpm; values in the figure are mean of triplicates ± SE, *** (*p* < 0.001).

Time-kill kinetics against *S. aureus* demonstrated clear differences between free ampicillin and nanocomposite-loaded formulations (Figure 7). During the first 3 h, free ampicillin produced slightly higher bacterial inhibition (≈45%) than the nanocomposites (≈40–44%), consistent with the initial lag phase associated with the release of the encapsulated drug. However, after 6 h, the nanocomposite formulations surpassed free ampicillin, achieving ≈64–65% inhibition compared with ≈63% for the free drug, indicating a more sustained bactericidal profile. By 12 h, most formulations reached maximal inhibition (≈92–93%), except M100 + Amp, which exhibited significantly lower activity (≈87%). Importantly, prolonged incubation up to 48 h showed a gradual decline in the antibacterial efficacy of free ampicillin (from 92% to 88%), reflecting the known resilience of the *S. aureus* strain and the reduced stability of free β-lactam antibiotics. In contrast, chitosan/CMC nanocomposites, particularly C3M1 + Amp and C2M1 + Amp, maintained stable inhibition (~92.2%) throughout 48 h, and delayed loss of activity compared to free ampicillin. The differences among groups were statistically significant (*p* < 0.001).

Overall, these findings indicate that ampicillin encapsulated in chitosan/CMC nanocomposites, benefiting from higher encapsulation efficiency and a slower, sustained release profile, delivers markedly enhanced antibacterial performance compared with the free drug and with previously reported chitosan-based delivery systems [22,23,27,37,38] (Table 3).

#### 3.2.3. MIC and MBC Evaluation

The MIC and minimum bactericidal concentration (MBC) values of ampicillin-loaded chitosan/cellulose nanocomposites are summarized in Table 2. The MIC and MBC values ranged from 3.20 to 3.47 µg/mL and 5.33–5.80 µg/mL, respectively, which are significantly lower than those of free ampicillin (4.23 and 6.80 µg/mL), C100 + Amp, and M100 + Amp. Moreover, the MBC/MIC ratios of chitosan/CMC nanocomposites (1.66–1.70) were lower than those of chitosan nanoparticles (C100) and carboxymethyl cellulose (M100)-loaded ampicillin, indicating improved bactericidal efficiency.

This study demonstrates that spray-dried chitosan/CMC nanocomposites constitute an efficient antibiotic delivery system with enhanced physicochemical and antibacterial performance. The comparison of chitosan/carboxymethyl cellulose that encapsulated ampicillin to other nanocarriers were shown in Table 3.

First, the formation of stable polyelectrolyte nanocomposites was achieved through electrostatic complexation between cationic chitosan and anionic CMC. Similar polyelectrolyte interactions have been reported to improve particle stability and functional performance in polysaccharide-based nanocarriers [39,40,41]. However, most previous systems relied on chemical crosslinkers or ionic gelation agents such as tripolyphosphate (TPP), which may limit scalability and biocompatibility [16,25,37].

Second, the nanocomposites exhibited exceptionally high positive zeta potentials (+70 to +90 mV) at optimal chitosan/CMC ratios. These values are significantly higher than those commonly reported for chitosan nanoparticles prepared by ionic gelation or spray drying (+20 to +55 mV). Recent studies have highlighted that increased surface charge enhances colloidal stability and bacterial membrane interaction, thereby improving antimicrobial efficacy [16,20,28].

Third, the ampicillin encapsulation efficiency reached approximately 80–82%, exceeding values reported for chitosan only or chitosan/starch systems (typically 60–75%) [21,24]. This improvement is attributed to the dense ionic network formed between oppositely charged polymers, which has been shown to enhance drug retention and loading capacity in polyelectrolyte-based carriers.

Fourth, ampicillin-loaded chitosan/CMC nanocomposites showed sustained and enhanced antibacterial activity against *Staphylococcus aureus*, maintaining inhibitory efficacy for up to 48 h. In contrast, free ampicillin exhibited a time-dependent decrease in activity, consistent with the reported resistance profile of *S. aureus* strains [21,29]. Similar sustained antibacterial effects have been observed in nano-enabled antibiotic delivery systems that protect β-lactam antibiotics from enzymatic degradation and efflux mechanisms [3,5,13,29,30,34].

Finally, the significantly reduced MIC and MBC values of the nanocomposites compared with free ampicillin and control nanoparticles confirm improved bacteriostatic and bactericidal performance. Previous studies have demonstrated that nanoencapsulation can lower MIC values by enhancing local drug concentration at the bacterial surface and disrupting cell membrane integrity (Table 3) [27,37,38].

Among all formulations, C2M1 exhibited the most favorable characteristics, including a mean particle size of 1288.8 nm, a high surface charge (+90.37 mV), a yield of 71.1%, and an encapsulation efficiency of 82.4%. It also demonstrated a more uniform size distribution (low PDI = 0.52) and good ampicillin release behavior. The C2M1 + Amp formulation showed the highest and most sustained antibacterial activity against *S. aureus* over 48 h, with the lowest MIC/MBC values (3.20/5.33) among all the tested samples. Therefore, C2M1 is considered the optimal formulation for the intended application.

## 4. Conclusions

Electrolyte gelation combined with spray drying provides a robust and efficient strategy for fabricating chitosan/carboxymethyl cellulose nanocomposites as antibiotic delivery carriers. The synergistic interaction between chitosan and CMC facilitated the formation of stable nanocomposites, exhibiting a high surface charge, superior ampicillin encapsulation efficiency, and sustained antibacterial activity against *Staphylococcus aureus*. The markedly reduced MIC and MBC values, together with prolonged antibacterial effectiveness, clearly demonstrate the ability of this delivery system to enhance antibiotic performance and potentially mitigate bacterial resistance.

Overall, these findings highlight chitosan/carboxymethyl cellulose nanocomposites as a promising, safe, and cost-effective platform for antimicrobial applications, providing a solid foundation for further preclinical evaluation and translational development in drug delivery and infection control.

## Figures and Tables

**Table 1:** Effect of chitosan/carboxymethyl cellulose ratio on the properties of chitosan/cellulose nanocomposites prepared by spray drying.

No	Samples	Chitosan/CMC Ratio (*w*/*w*)	Zeta Potential (mV)	Mean Particle Size (nm)	Polydispersity Index (PDI)	Yield (%)	Encapsulation Efficiency of Ampicillin (%)
1	C100	1:0	67.67 ± 0.92	816.9 ± 24.5 ^e^	0.67 ± 0.04	75.49 ± 0.35 ^a^	68.6 ± 1.2 ^c^
2	C5M1	5:1	82.70 ± 0.45	954.2 ± 20.3 ^e^	0.48 ± 0.02	72.03 ± 0.06 ^b^	78.8 ± 0.9 ^b^
3	C4M1	4:1	85.07 ± 1.81	1049.8 ± 28.7 ^d^	0.65 ± 0.04	71.88 ± 0.07 ^b^	79.2 ± 1.3 ^b^
4	C3M1	3:1	89.53 ± 1.89	1144.4 ± 32.0 ^d^	0.53 ± 0.02	71.17 ± 0.04 ^b^	81.8 ± 0.7 ^a^
5	C2M1	2:1	90.37 ± 1.05	1288.8 ± 24.3 ^c^	0.52 ± 0.02	71.08 ± 0.03 ^b^	82.4 ± 1.4 ^a^
6	C1M1	1:1	78.63 ± 1.02	1290.4 ± 35.0 ^c^	0.66 ± 0.02	71.04 ± 0.05 ^b^	80.1 ± 0.9 ^b^
7	C1M2	1:2	61.67 ± 2.45	1304.7 ± 26.3 ^c^	1.03 ± 0.06	70.71 ± 0.06 ^c^	75.8 ± 1.9 ^c^
8	C1M3	1:3	−31.43 ± 0.65	1313.0 ± 18.2 ^c^	0.60 ± 0.07	69.15 ± 0.03 ^d^	58.6 ± 0.7 ^d^
9	C1M4	1:4	−39.17 ± 0.05	1652.5 ± 42.5 ^b^	0.75 ± 0.04	69.06 ± 0.04 ^d^	56.3 ± 0.4 ^d^
10	C1M5	1:5	−40.00 ± 0.72	1906.8 ± 40.4 ^a^	1.00 ± 0.01	68.72 ± 0.60 ^e^	50.8 ± 0.8 ^e^
11	M100	0:1	−81.73 ± 0.90	1658.9 ± 38.7 ^b^	0.64 ± 0.01	67.01 ± 0.06 ^e^	38.8 ± 0.6 ^f^

Values are presented as mean ± standard deviation (n = 3). Different superscript letters (a–f) within the same column indicate statistically significant differences (*p* < 0.05). C100: Data of this sample is from the paper [21].

**Table 2:** MIC and MBC of chitosan/carboxymethyl cellulose nanocomposite with loaded ampicillin on *Staphyllococcus aureus*.

No.	Samples	MIC (µg/mL)	MBC (µg/mL)	MBC/MIC Ratio
1	C100 + Amp	3.53 ^c^ ± 0.12	6.47 ^c^ ± 0.12	1.83
2	C2M1 + Amp	3.20 ^a^ ± 0.00	5.33 ^a^ ± 0.12	1.66
3	C1M1 + Amp	3.30 ^ab^ ± 0.12	5.73 ^b^ ± 0.12	1.70
4	C1M2 + Amp	3.47 ^c^ ± 0.12	5.80 ^b^ ± 0.00	1.67
5	M100 + Amp	4.27 ^d^ ± 0.12	7.07 ^e^ ± 0.12	1.65
6	Ampicillin (Amp)	4.23 ^d^ ± 0.12	6.80 ^d^ ± 0.00	1.60

Values are presented as mean ± standard deviation (n = 3). Different superscript letters (a–e) within the same column indicate statistically significant differences (*p* < 0.05).

**Table 3:** The comparison of chitosan/carboxymethyl cellulose nanocomposite that encapsulated ampicillin to other nanocarriers.

Systems	EE%	MIC (µg/mL)	MBC (µg/mL)	Key Notes	References
C100 + Amp	68.6	3.53	6.47	Lower EE%, and lower activity	This study
C2M1 + Amp	82.4	3.20	5.33	Highest EE%; lowest MIC/MBC; strongest bactericidal efficiency	This study
M100 + Amp	38.8	4.27	7.07	Lowest antibacterial performance	This study
Ampicillin (Amp)	-	4.23	6.80	Decline in activity at 24–48 h	This study
Chitosan nanoparticles + Amp (spray drying)	-	8–10	-	Lower activity	[20]
Chitosan nano-microparticles + Amp (ionic gelation)	-	8.0–12.5	8.0–12.0	Moderate activity, limited EE%	[23,27,37,38]
Chitosan/starch nanocomposite + Amp	75–77	4.0–5.0	9.0–10.0	Higher MIC; weaker polymer–drug interaction	[21,24]
Other nano β-lactam carriers	50–60	3.5–6.0	6.0–12.0	Improved stability but require chemical crosslinkers	[29,30,36]

## Data Availability

The original contributions presented in this study are included in the article. Further inquiries can be directed to the corresponding author.

## Supplementary Materials

The following supporting information can be downloaded at: https://www.mdpi.com/article/10.3390/polym18030319/s1, Figure S1: Zeta potential of the nanocomposites; Figure S2: Size distribution; Figure S3: FT-IR of the nanocomposite and nanocomposite loaded ampicillin.
