# Supplementary material for: Chitosan/Carboxymethyl Cellulose Nanocomposites Prepared via Electrolyte Gelation–Spray Drying for Controlled Ampicillin Delivery and Enhanced Antibacterial Activity
Source: Polymers (Basel). 2026 Jan 24;18(3):319. doi: 10.3390/polym18030319 (PMC12899446; doi:10.3390/polym18030319)

HORIBA Scientific

HORIBA SZ-100 for Windows [Z Type] Ver2.50

SZ-100

Measurement Results

Measurement Results

Date	:	Saturday, May 6, 2023 4:58:08 PM
Measurement Type	:	Zeta Potential
Sample Name	:	C1M1
Temperature of the Holder	:	25.0 °C
Dispersion Medium Viscosity	:	0.895 mPa·s
Conductivity	:	0.443 mS/cm
Electrode Voltage	:	3.3 V

Calculation Results

Peak No.	Zeta Potential	Electrophoretic Mobility
1	79.8 mV	0.000618 cm2/Vs
2	--- mV	--- cm2/Vs
3	--- mV	--- cm2/Vs

Zeta Potential (Mean)	:	79.8 mV
Electrophoretic Mobility Mean	:	0.000618 cm^2/Vs

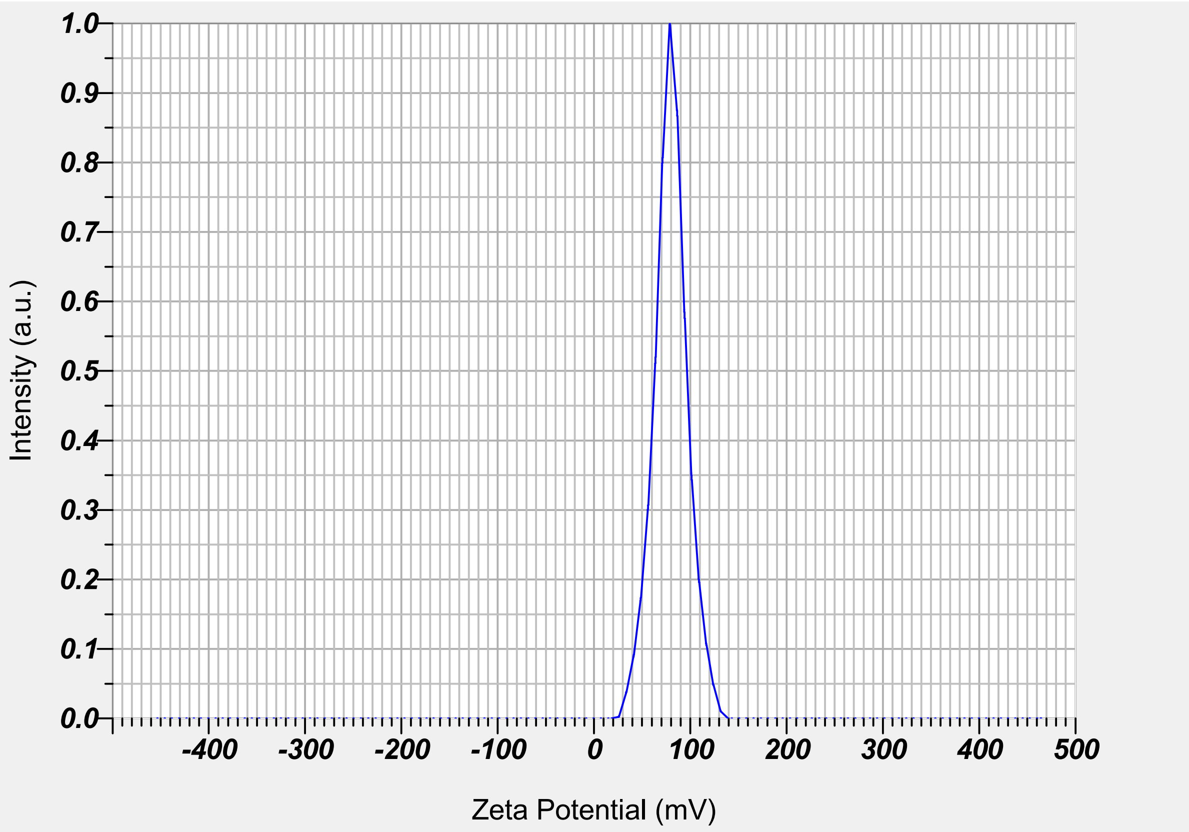

HORIBA Scientific

HORIBA SZ-100 for Windows [Z Type] Ver2.50

SZ-100

Measurement Results

Measurement Results

Date	:	Saturday, May 6, 2023 5:10:37 PM
Measurement Type	:	Zeta Potential
Sample Name	:	C1M2
Temperature of the Holder	:	25.0 °C
Dispersion Medium Viscosity	:	0.896 mPa·s
Conductivity	:	0.413 mS/cm
Electrode Voltage	:	3.3 V

Calculation Results

Peak No.	Zeta Potential	Electrophoretic Mobility
1	64.5 mV	0.000499 cm2/Vs
2	--- mV	--- cm2/Vs
3	--- mV	--- cm2/Vs

Zeta Potential (Mean)	:	64.5 mV
Electrophoretic Mobility Mean	:	0.000499 cm^2/Vs

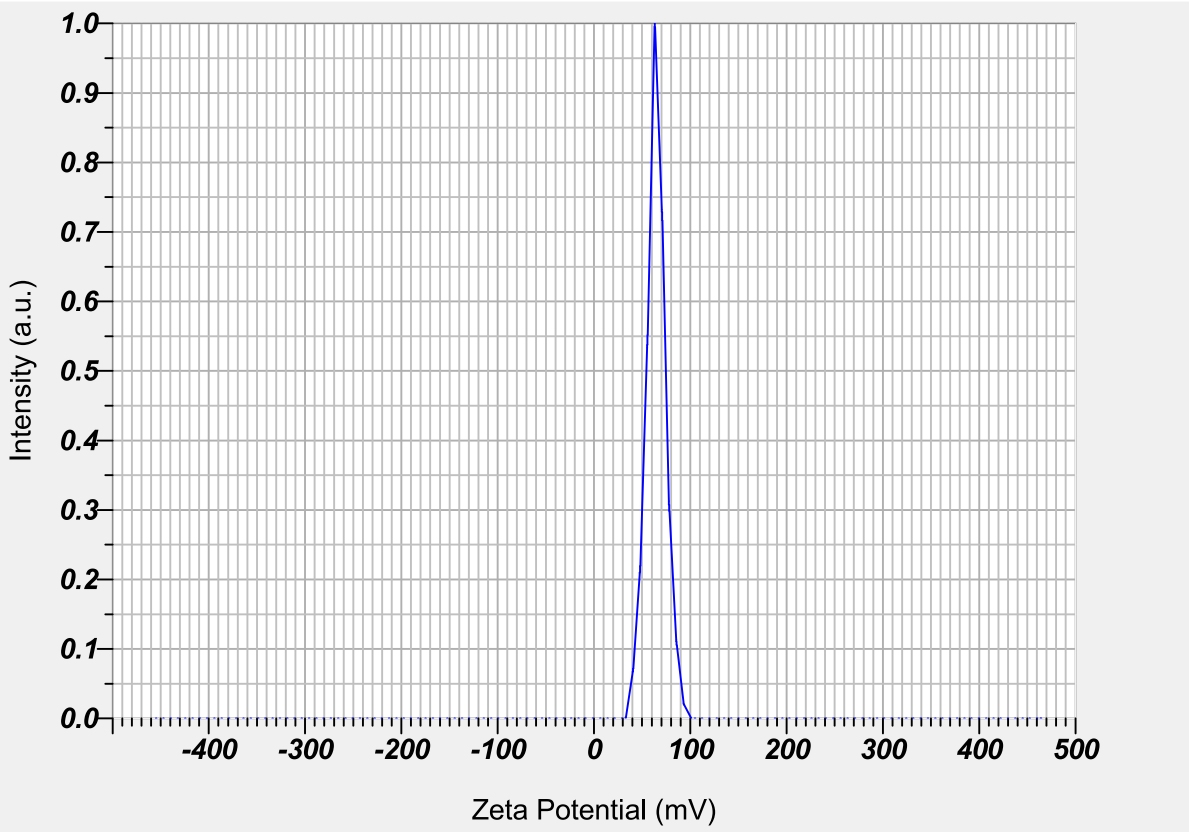

HORIBA Scientific

HORIBA SZ-100 for Windows [Z Type] Ver2.50

SZ-100

Measurement Results

Measurement Results

Date	:	Saturday, May 6, 2023 5:15:09 PM
Measurement Type	:	Zeta Potential
Sample Name	:	C1M3
Temperature of the Holder	:	24.9 °C
Dispersion Medium Viscosity	:	0.897 mPa·s
Conductivity	:	0.409 mS/cm
Electrode Voltage	:	3.3 V

Calculation Results

Peak No.	Zeta Potential	Electrophoretic Mobility
1	-31.4 mV	-0.000242 cm2/Vs
2	--- mV	--- cm2/Vs
3	--- mV	--- cm2/Vs

Zeta Potential (Mean)	:	-31.4 mV
Electrophoretic Mobility Mean	:	-0.000242 cm^2/Vs

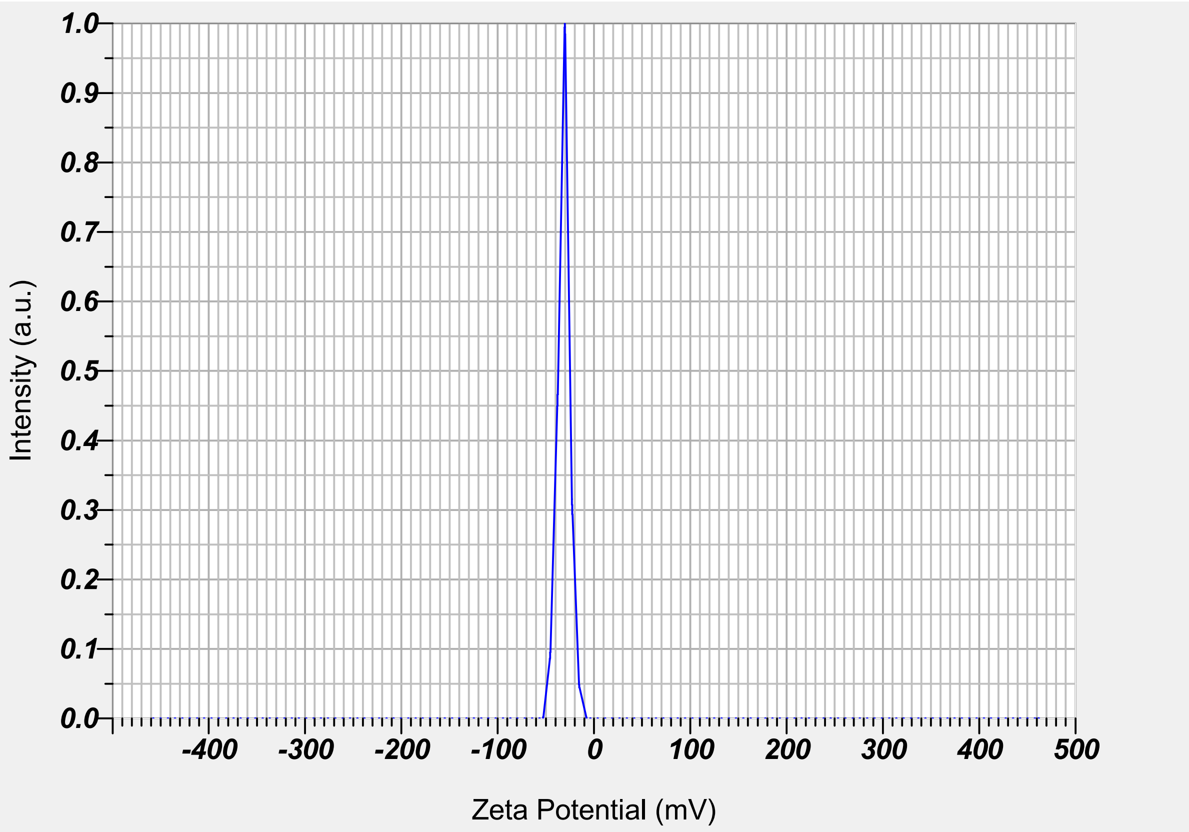

HORIBA Scientific

HORIBA SZ-100 for Windows [Z Type] Ver2.50

SZ-100

Measurement Results

Measurement Results

Date	:	Saturday, May 6, 2023 5:29:24 PM
Measurement Type	:	Zeta Potential
Sample Name	:	C1M4
Temperature of the Holder	:	25.0 °C
Dispersion Medium Viscosity	:	0.896 mPa·s
Conductivity	:	0.406 mS/cm
Electrode Voltage	:	3.3 V

Calculation Results

Peak No.	Zeta Potential	Electrophoretic Mobility
1	-39.2 mV	-0.000303 cm2/Vs
2	--- mV	--- cm2/Vs
3	--- mV	--- cm2/Vs

Zeta Potential (Mean)	:	-39.2 mV
Electrophoretic Mobility Mean	:	-0.000303 cm^2/Vs

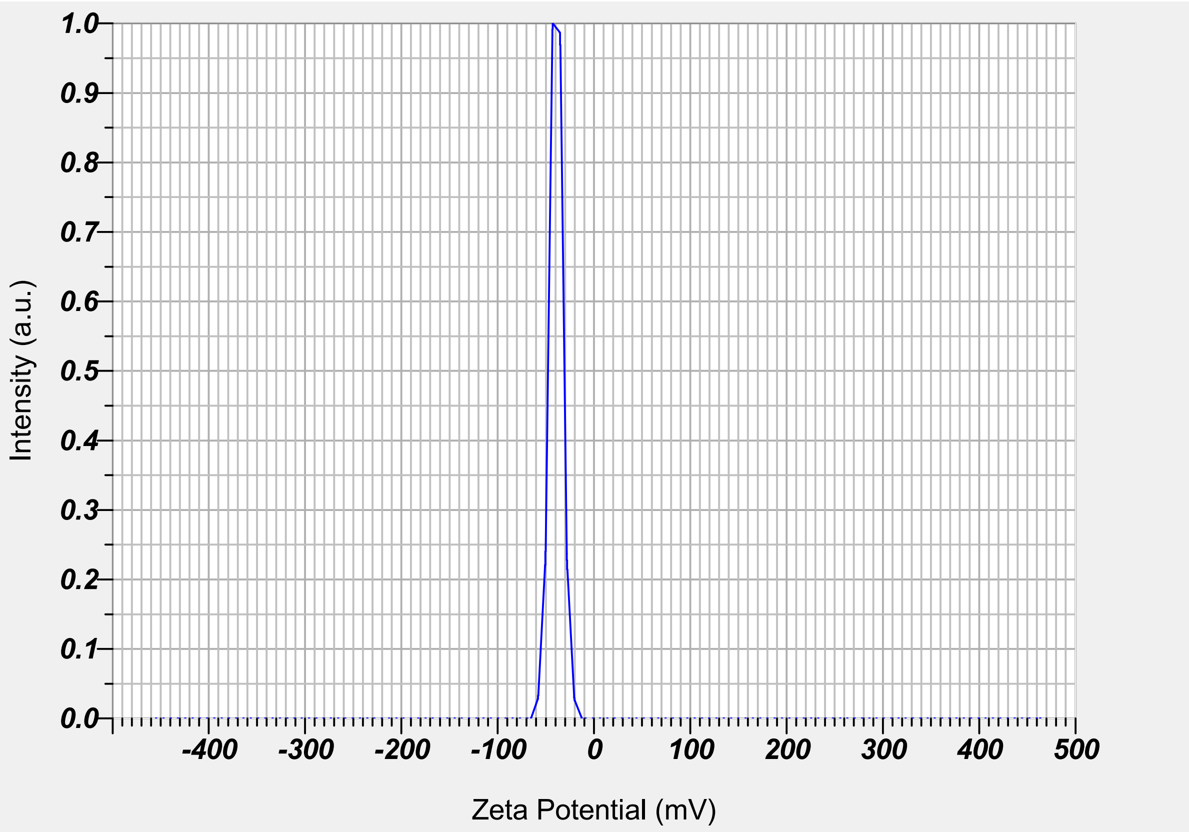

SZ-100

Measurement Results

Measurement Results

Date	:	Saturday, May 6, 2023 5:34:47 PM
Measurement Type	:	Zeta Potential
Sample Name	:	C1M5
Temperature of the Holder	:	25.0 °C
Dispersion Medium Viscosity	:	0.896 mPa·s
Conductivity	:	0.398 mS/cm
Electrode Voltage	:	3.3 V

Calculation Results

Peak No.	Zeta Potential	Electrophoretic Mobility
1	-40.2 mV	-0.000311 cm2/Vs
2	--- mV	--- cm2/Vs
3	--- mV	--- cm2/Vs

Zeta Potential (Mean)	:	-40.2 mV
Electrophoretic Mobility Mean	:	-0.000311 cm^2/Vs

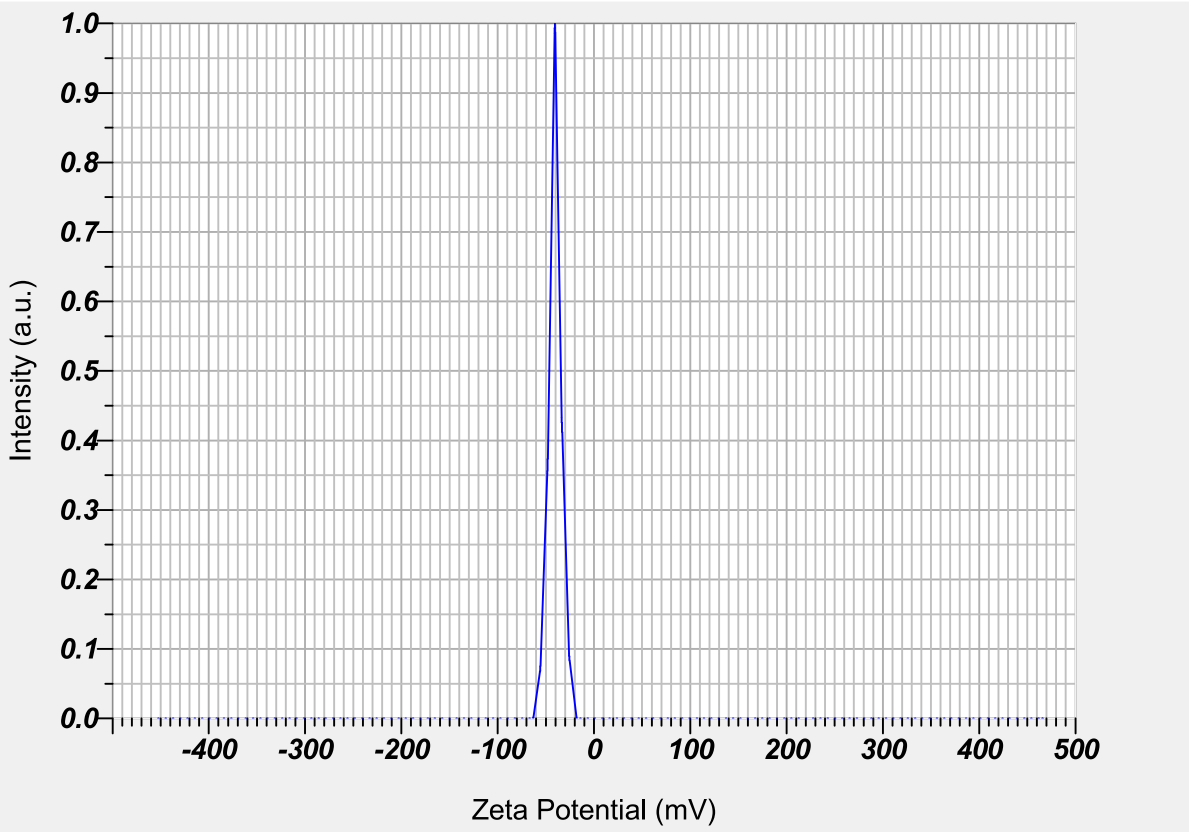

HORIBA Scientific

HORIBA SZ-100 for Windows [Z Type] Ver2.50

SZ-100

Measurement Results

Measurement Results

Date	:	Saturday, May 6, 2023 4:51:45 PM
Measurement Type	:	Zeta Potential
Sample Name	:	C2M1
Temperature of the Holder	:	24.9 °C
Dispersion Medium Viscosity	:	0.897 mPa·s
Conductivity	:	0.475 mS/cm
Electrode Voltage	:	3.3 V

Calculation Results

Peak No.	Zeta Potential	Electrophoretic Mobility
1	89.3 mV	0.000690 cm2/Vs
2	--- mV	--- cm2/Vs
3	--- mV	--- cm2/Vs

Zeta Potential (Mean)	:	89.3 mV
Electrophoretic Mobility Mean	:	0.000690 cm^2/Vs

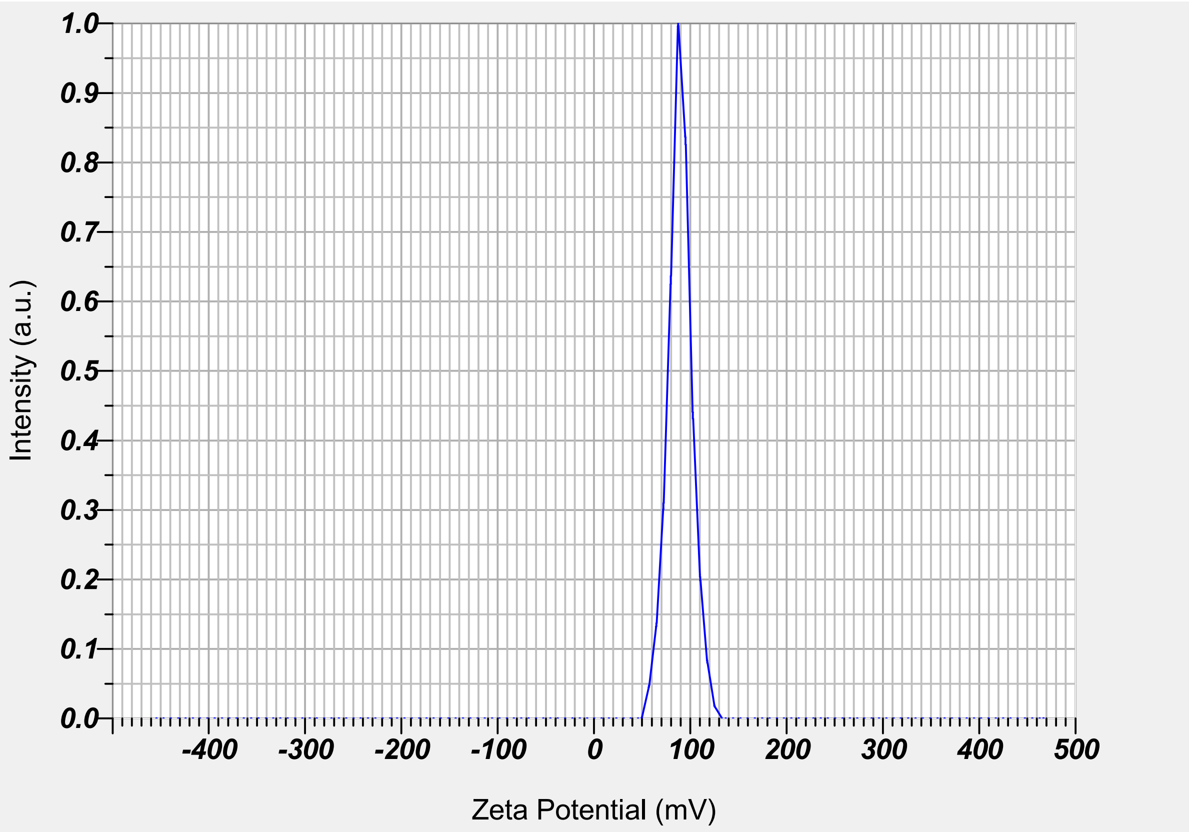

HORIBA Scientific

SZ-100

Measurement Results

Measurement Results

Date	:	Saturday, May 6, 2023 4:20:32 PM
Measurement Type	:	Zeta Potential
Sample Name	:	C4M1
Temperature of the Holder	:	25.0 °C
Dispersion Medium Viscosity	:	0.896 mPa·s
Conductivity	:	0.510 mS/cm
Electrode Voltage	:	3.3 V

Calculation Results

Peak No.	Zeta Potential	Electrophoretic Mobility
1	87.0 mV	0.000673 cm2/Vs
2	--- mV	--- cm2/Vs
3	--- mV	--- cm2/Vs

Zeta Potential (Mean)	:	87.0 mV
Electrophoretic Mobility Mean	:	0.000673 cm^2/Vs

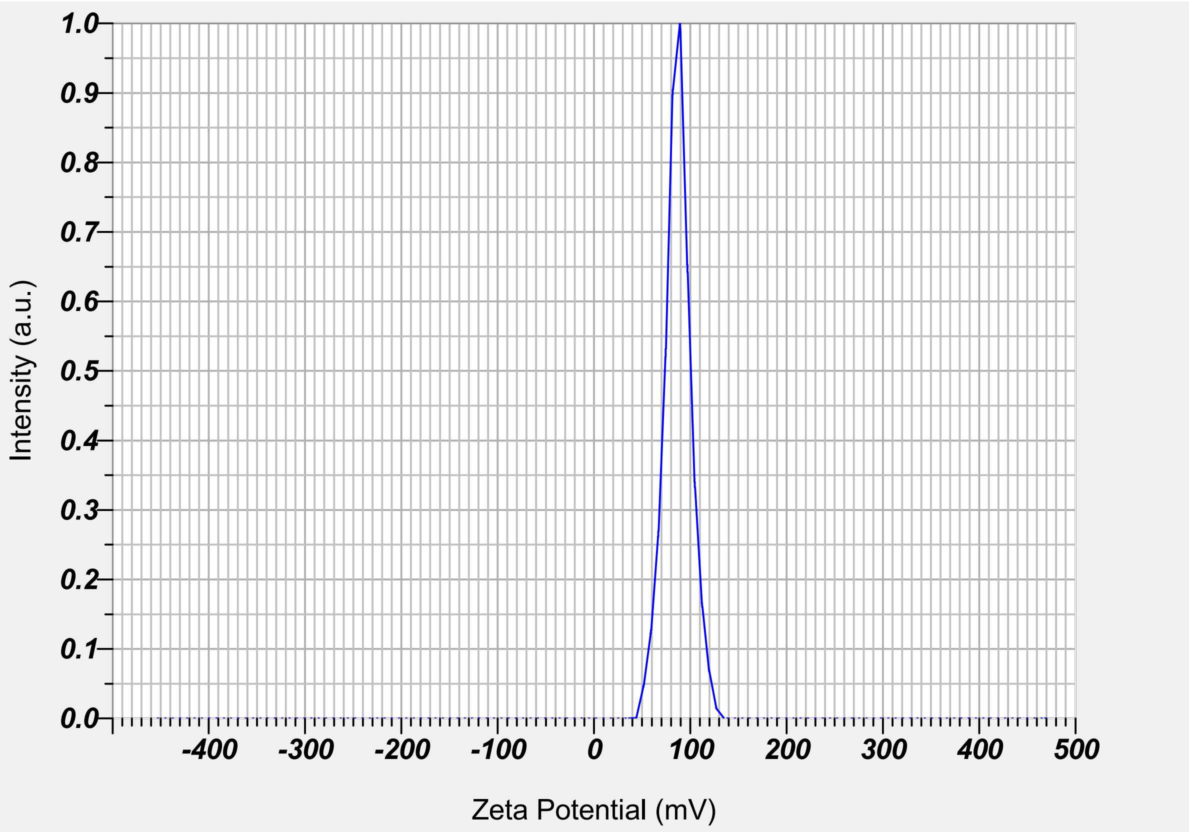

HORIBA Scientific

HORIBA SZ-100 for Windows [Z Type] Ver2.50

SZ-100

Measurement Results

Measurement Results

Date	:	Saturday, May 6, 2023 4:36:48 PM
Measurement Type	:	Zeta Potential
Sample Name	:	C3M1
Temperature of the Holder	:	24.8 °C
Dispersion Medium Viscosity	:	0.898 mPa·s
Conductivity	:	0.489 mS/cm
Electrode Voltage	:	3.3 V

Calculation Results

Peak No.	Zeta Potential	Electrophoretic Mobility
1	88.7 mV	0.000685 cm2/Vs
2	--- mV	--- cm2/Vs
3	--- mV	--- cm2/Vs

Zeta Potential (Mean)	:	88.7 mV
Electrophoretic Mobility Mean	:	0.000685 cm^2/Vs

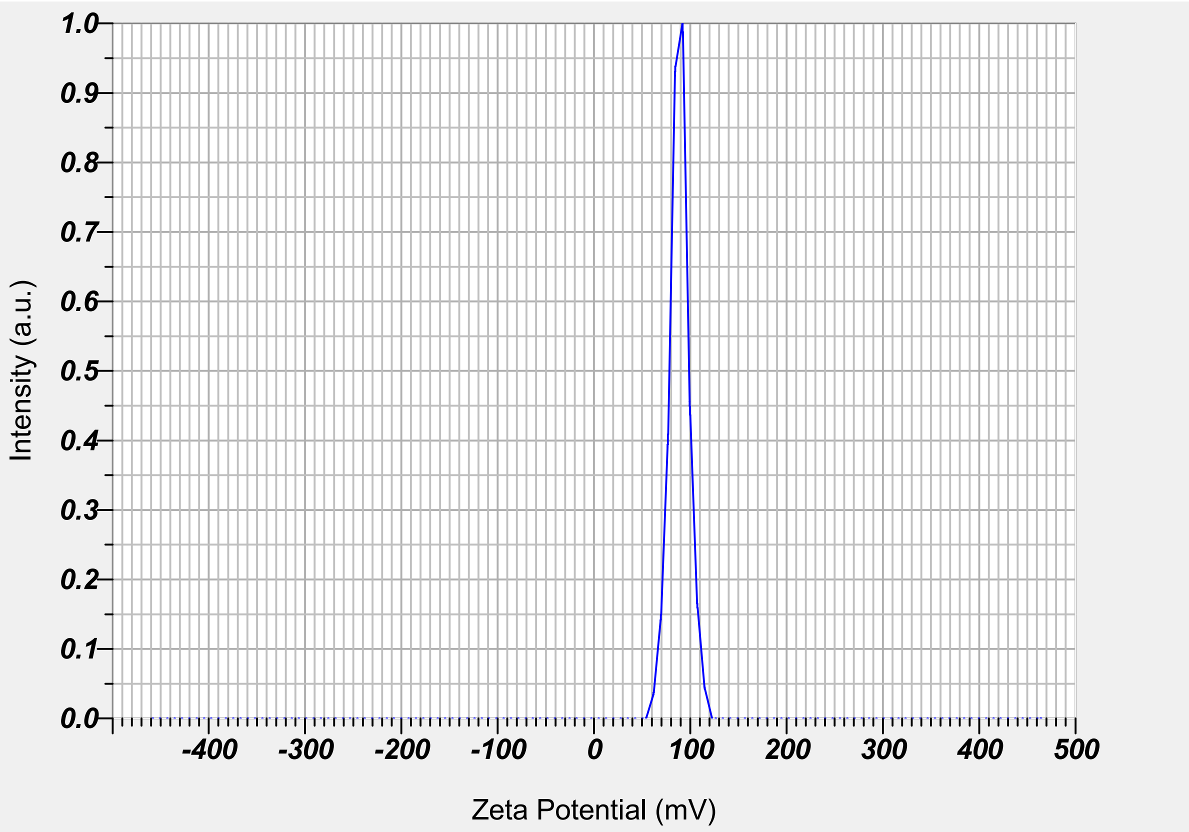

HORIBA Scientific

HORIBA SZ-100 for Windows [Z Type] Ver2.50

SZ-100

Measurement Results

Measurement Results

Date	:	Saturday, May 6, 2023 4:41:02 PM
Measurement Type	:	Zeta Potential
Sample Name	:	C5M1
Temperature of the Holder	:	25.0 °C
Dispersion Medium Viscosity	:	0.895 mPa·s
Conductivity	:	0.512 mS/cm
Electrode Voltage	:	3.3 V

Calculation Results

Peak No.	Zeta Potential	Electrophoretic Mobility
1	83.2 mV	0.000645 cm2/Vs
2	--- mV	--- cm2/Vs
3	--- mV	--- cm2/Vs

Zeta Potential (Mean)	:	83.2 mV
Electrophoretic Mobility Mean	:	0.000645 cm^2/Vs

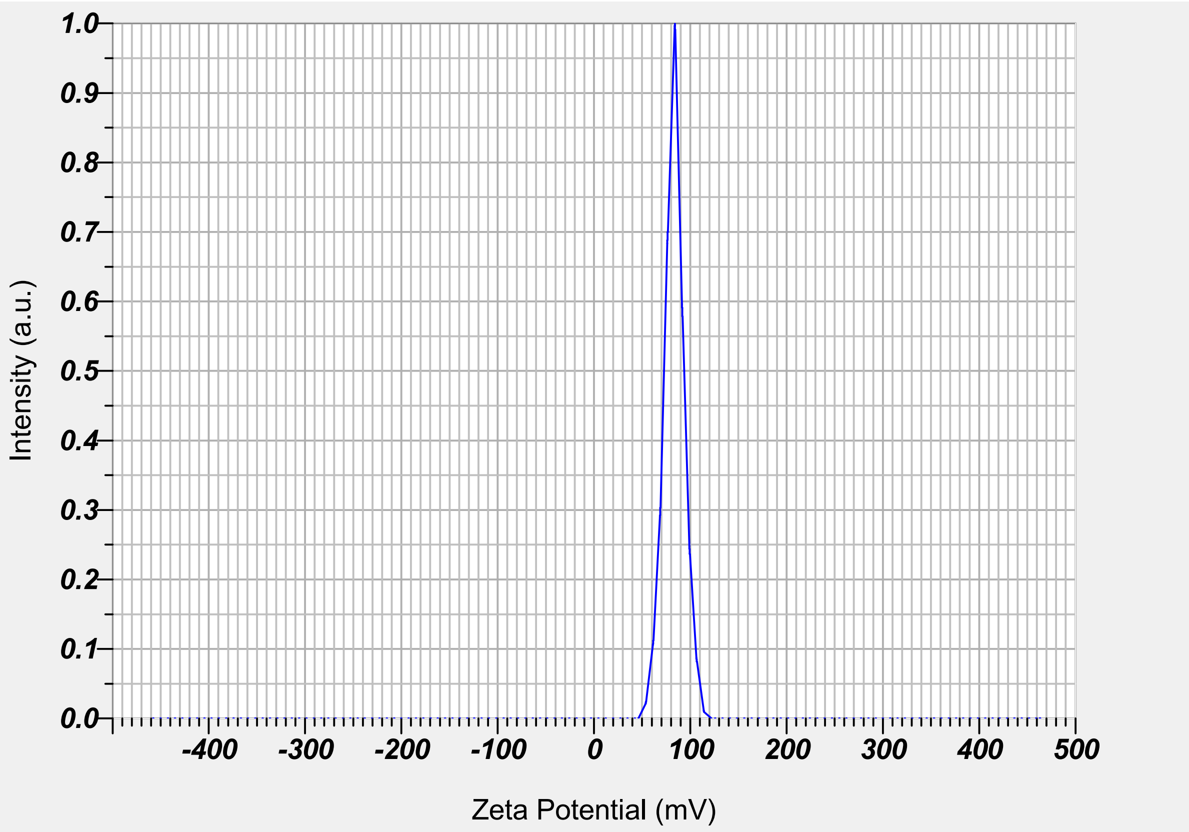

HORIBA Scientific

HORIBA SZ-100 for Windows [Z Type] Ver2.50

SZ-100

Measurement Results

Measurement Results

Date	:	Saturday, May 6, 2023 4:32:14 PM
Measurement Type	:	Zeta Potential
Sample Name	:	C100
Temperature of the Holder	:	25.0 °C
Dispersion Medium Viscosity	:	0.895 mPa·s
Conductivity	:	0.402 mS/cm
Electrode Voltage	:	3.3 V

Calculation Results

Peak No.	Zeta Potential	Electrophoretic Mobility
1	68.3 mV	0.000529 cm2/Vs
2	--- mV	--- cm2/Vs
3	--- mV	--- cm2/Vs

Zeta Potential (Mean) : 68.3 mV

Electrophoretic Mobility Mean : 0.000529 cm^2/Vs

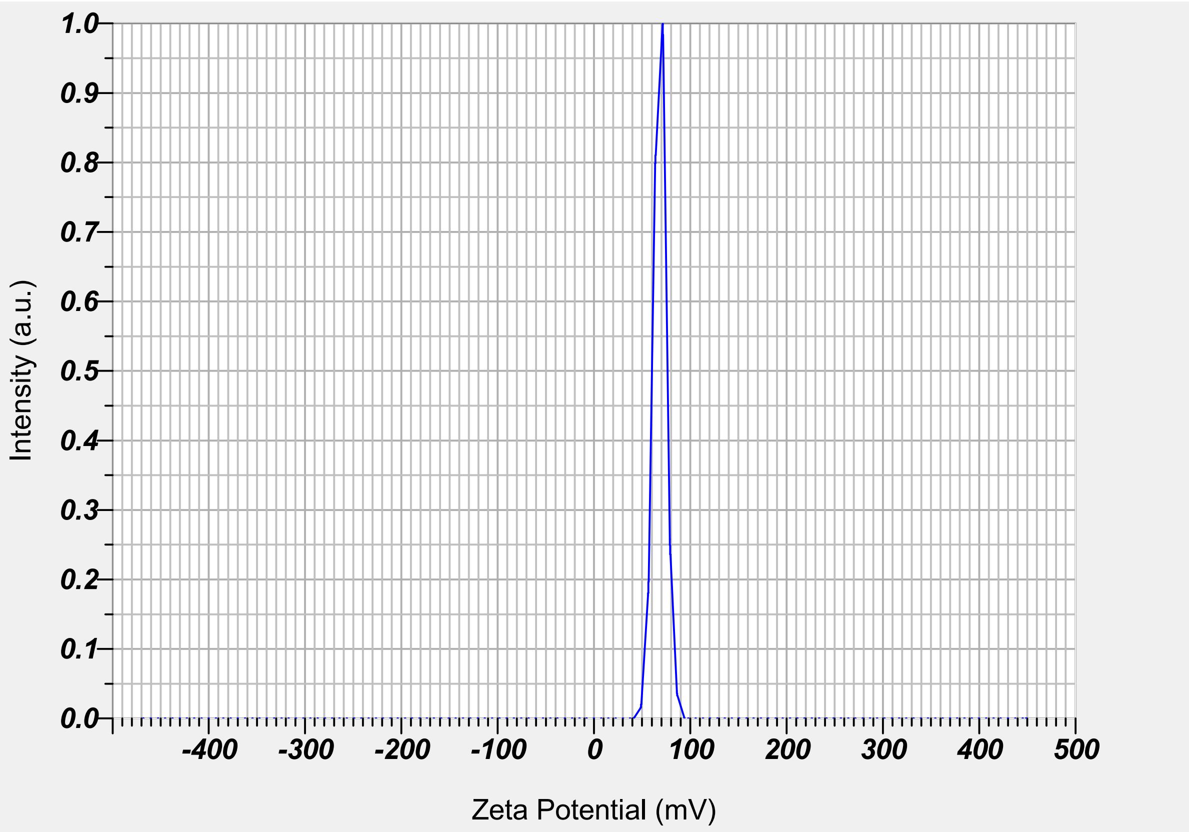

HORIBA Scientific

SZ-100

Measurement Results

Measurement Results

Date : Saturday, May 6, 2023 5:39:15 PM
Measurement Type : Zeta Potential
Sample Name : M100
Temperature of the Holder : 25.0 °C
Dispersion Medium Viscosity : 0.895 mPa·s
Conductivity : 0.365 mS/cm
Electrode Voltage : 3.3 V

Calculation Results

Peak No.	Zeta Potential	Electrophoretic Mobility
1	-80.7 mV	-0.000625 cm2/Vs
2	--- mV	--- cm2/Vs
3	--- mV	--- cm2/Vs

Zeta Potential (Mean) : -80.7 mV
Electrophoretic Mobility Mean : -0.000625 cm^2/Vs

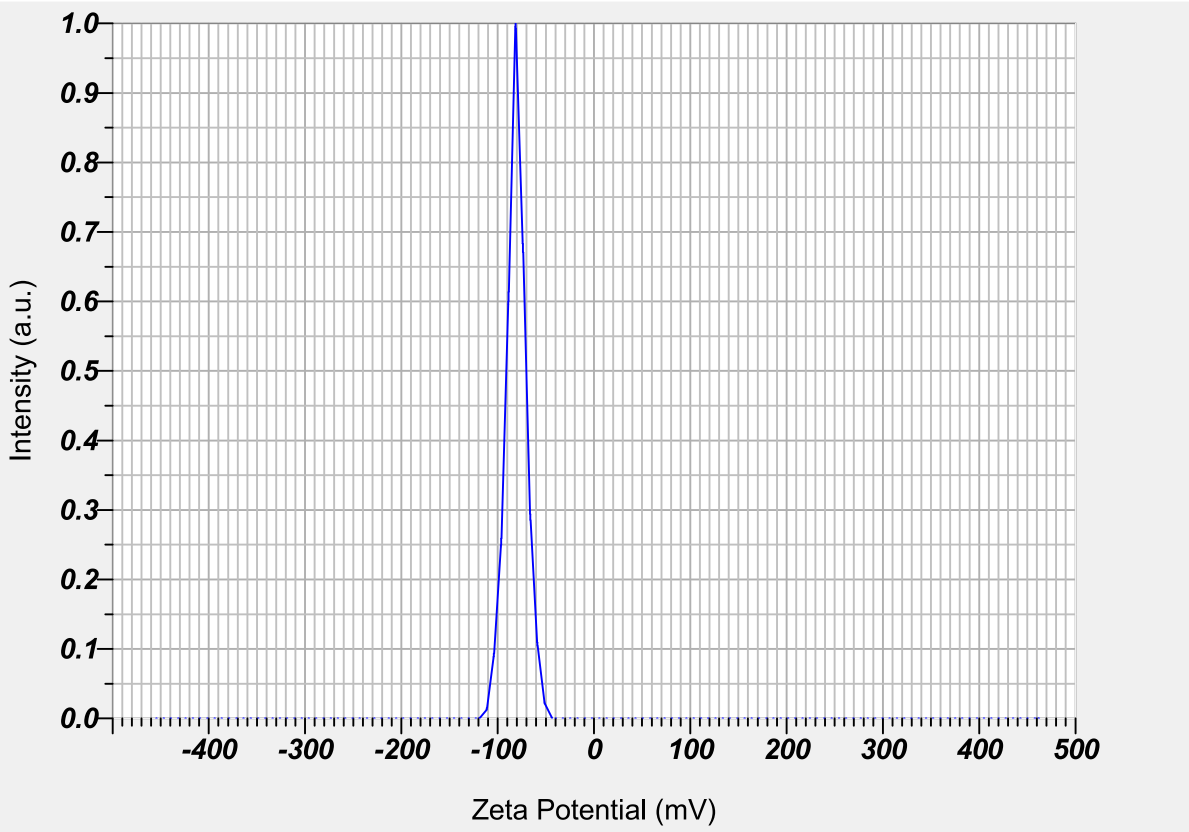

Supplement: Supplementary file 1 [file polymers-18-00319-s001.zip › Figure S1. Zeta potential of the nanocomposites (2).pdf]
