# Supplementary material for: Chitosan/Carboxymethyl Cellulose Nanocomposites Prepared via Electrolyte Gelation–Spray Drying for Controlled Ampicillin Delivery and Enhanced Antibacterial Activity
Source: Polymers (Basel). 2026 Jan 24;18(3):319. doi: 10.3390/polym18030319 (PMC12899446; doi:10.3390/polym18030319)
Supplement: Supplementary file 1 [file polymers-18-00319-s001.zip › Figure S2. Size distribution of the nanocomposites PDF (1).pdf]

SZ-100

C1M1.nsz

Measurement Results

Date	:	Monday, May 8, 2023 7:05:32 PM
Measurement Type	:	Particle Size
Sample Name	:	C1M1
Scattering Angle	:	90
Temperature of the Holder	:	25.0 °C
Dispersion Medium Viscosity	:	0.895 mPa·s
Transmission Intensity before Meas.	:	24954
Distribution Form	:	Standard
Distribution Form(Dispersity)	:	Monodisperse
Representation of Result	:	Scattering Light Intensity
Count Rate	:	15 kCPS

Calculation Results

Peak No.	S.P.Area Ratio	Mean	S. D.	Mode
1	1.00	855.7 nm	256.2 nm	784.9 nm
2	---	--- nm	--- nm	--- nm
3	---	--- nm	--- nm	--- nm
Total	1.00	855.7 nm	256.2 nm	784.9 nm

Cumulant Operations

Z-Average	:	1290.4 nm
PI	:	0.683

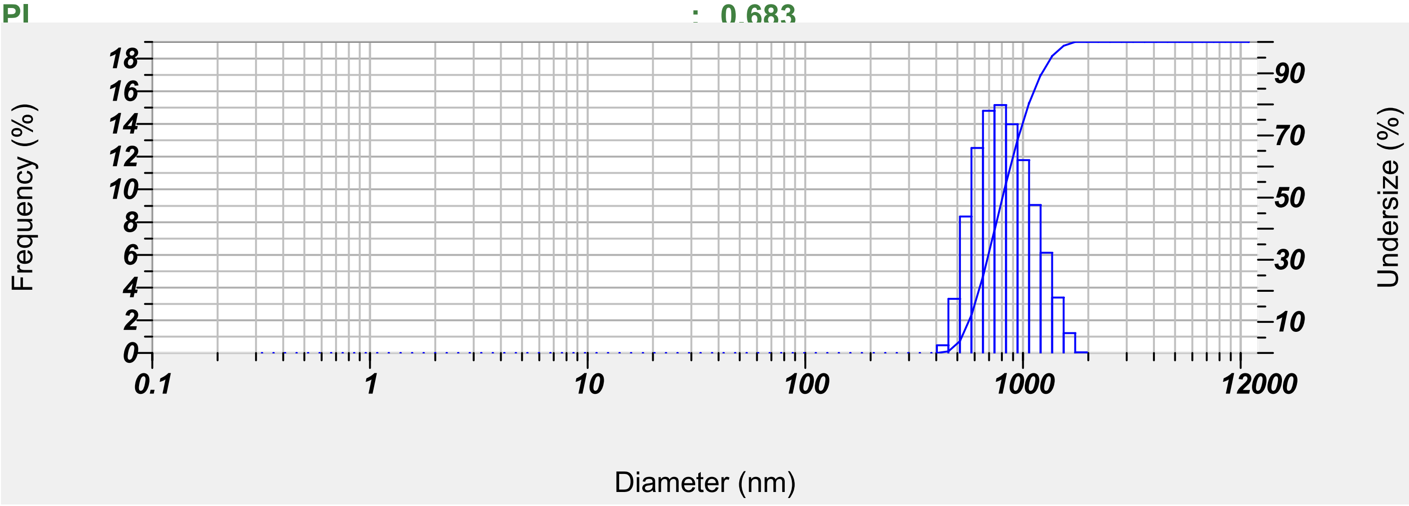

No.	Diameter	Frequency	Cumulation	No.	Diameter	Frequency	Cumulation	No.	Diameter	Frequency	Cumulation	No.	Diameter	Frequency	Cumulation
1	0.34	0.000	0.000	23	4.97	0.000	0.000	45	72.87	0.000	0.000	67	1068.52	11.779	80.240
2	0.38	0.000	0.000	24	5.61	0.000	0.000	46	82.33	0.000	0.000	68	1207.24	9.028	89.268
3	0.43	0.000	0.000	25	6.34	0.000	0.000	47	93.02	0.000	0.000	69	1363.97	6.111	95.379
4	0.49	0.000	0.000	26	7.17	0.000	0.000	48	105.10	0.000	0.000	70	1541.04	3.380	98.759
5	0.55	0.000	0.000	27	8.10	0.000	0.000	49	118.74	0.000	0.000	71	1741.10	1.205	99.963
6	0.62	0.000	0.000	28	9.15	0.000	0.000	50	134.16	0.000	0.000	72	1967.14	0.037	100.000
7	0.70	0.000	0.000	29	10.34	0.000	0.000	51	151.57	0.000	0.000	73	2222.51	0.000	100.000
8	0.80	0.000	0.000	30	11.68	0.000	0.000	52	171.25	0.000	0.000	74	2511.05	0.000	100.000
9	0.90	0.000	0.000	31	13.20	0.000	0.000	53	193.48	0.000	0.000	75	2837.04	0.000	100.000
10	1.02	0.000	0.000	32	14.91	0.000	0.000	54	218.60	0.000	0.000	76	3205.35	0.000	100.000
11	1.15	0.000	0.000	33	16.84	0.000	0.000	55	246.98	0.000	0.000	77	3621.48	0.000	100.000
12	1.30	0.000	0.000	34	19.03	0.000	0.000	56	279.04	0.000	0.000	78	4091.63	0.000	100.000
13	1.47	0.000	0.000	35	21.50	0.000	0.000	57	315.27	0.000	0.000	79	4622.81	0.000	100.000
14	1.66	0.000	0.000	36	24.29	0.000	0.000	58	356.20	0.000	0.000	80	5222.96	0.000	100.000
15	1.87	0.000	0.000	37	27.45	0.000	0.000	59	402.44	0.000	0.000	81	5901.02	0.000	100.000
16	2.11	0.000	0.000	38	31.01	0.000	0.000	60	454.69	0.454	0.454	82	6667.10	0.000	100.000
17	2.39	0.000	0.000	39	35.03	0.000	0.000	61	513.71	3.296	3.749	83	7532.65	0.000	100.000
18	2.70	0.000	0.000	40	39.58	0.000	0.000	62	580.41	8.326	12.076	84	8510.56	0.000	100.000
19	3.05	0.000	0.000	41	44.72	0.000	0.000	63	655.76	12.514	24.589	85	9615.42	0.000	100.000
20	3.45	0.000	0.000	42	50.53	0.000	0.000	64	740.89	14.779	39.368	86	10863.72	0.000	100.000
21	3.89	0.000	0.000	43	57.09	0.000	0.000	65	837.07	15.128	54.496				
22	4.40	0.000	0.000	44	64.50	0.000	0.000	66	945.74	13.965	68.461				

SZ-100

C1M2.nsz

Measurement Results

Date	:	Monday, May 8, 2023 6:57:57 PM
Measurement Type	:	Particle Size
Sample Name	:	C1M2
Scattering Angle	:	90
Temperature of the Holder	:	25.0 °C
Dispersion Medium Viscosity	:	0.895 mPa·s
Transmission Intensity before Meas.	:	25040
Distribution Form	:	Standard
Distribution Form(Dispersity)	:	Monodisperse
Representation of Result	:	Scattering Light Intensity
Count Rate	:	51 kCPS

Calculation Results

Peak No.	S.P.Area Ratio	Mean	S. D.	Mode
1	1.00	1754.9 nm	494.6 nm	1631.3 nm
2	---	--- nm	--- nm	--- nm
3	---	--- nm	--- nm	--- nm
Total	1.00	1754.9 nm	494.6 nm	1631.3 nm

Cumulant Operations

Z-Average	:	1304.7 nm
PI	:	1.100

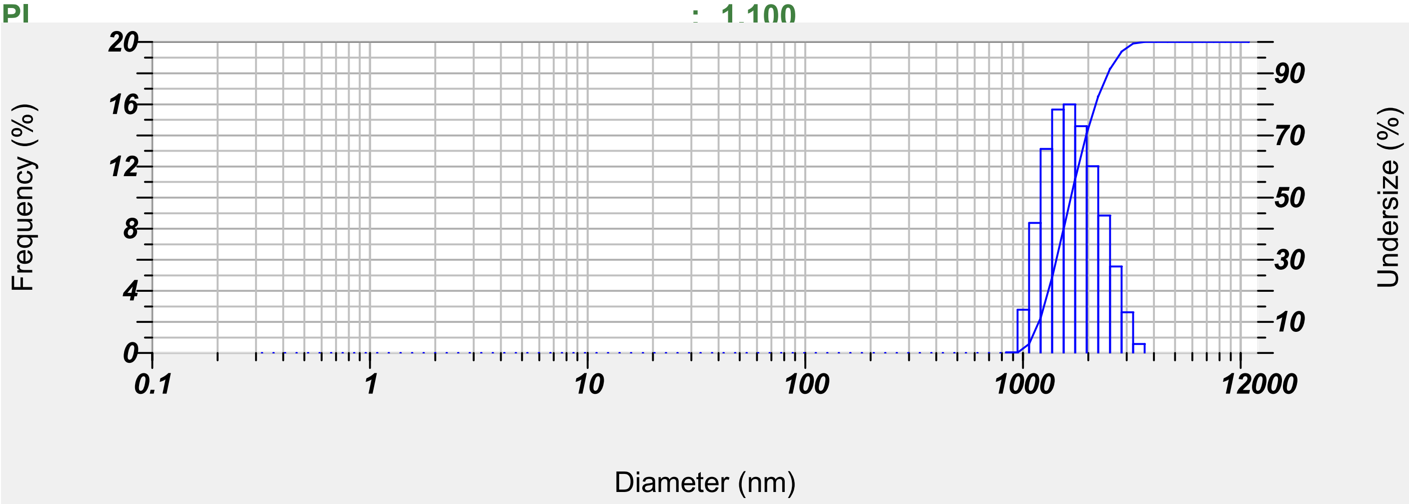

No.	Diameter	Frequency	Cumulation	No.	Diameter	Frequency	Cumulation	No.	Diameter	Frequency	Cumulation	No.	Diameter	Frequency	Cumulation
1	0.34	0.000	0.000	23	4.97	0.000	0.000	45	72.87	0.000	0.000	67	1068.52	2.767	2.790
2	0.38	0.000	0.000	24	5.61	0.000	0.000	46	82.33	0.000	0.000	68	1207.24	8.359	11.150
3	0.43	0.000	0.000	25	6.34	0.000	0.000	47	93.02	0.000	0.000	69	1363.97	13.104	24.253
4	0.49	0.000	0.000	26	7.17	0.000	0.000	48	105.10	0.000	0.000	70	1541.04	15.650	39.903
5	0.55	0.000	0.000	27	8.10	0.000	0.000	49	118.74	0.000	0.000	71	1741.10	15.980	55.884
6	0.62	0.000	0.000	28	9.15	0.000	0.000	50	134.16	0.000	0.000	72	1967.14	14.568	70.451
7	0.70	0.000	0.000	29	10.34	0.000	0.000	51	151.57	0.000	0.000	73	2222.51	11.997	82.448
8	0.80	0.000	0.000	30	11.68	0.000	0.000	52	171.25	0.000	0.000	74	2511.05	8.823	91.271
9	0.90	0.000	0.000	31	13.20	0.000	0.000	53	193.48	0.000	0.000	75	2837.04	5.541	96.812
10	1.02	0.000	0.000	32	14.91	0.000	0.000	54	218.60	0.000	0.000	76	3205.35	2.615	99.427
11	1.15	0.000	0.000	33	16.84	0.000	0.000	55	246.98	0.000	0.000	77	3621.48	0.573	100.000
12	1.30	0.000	0.000	34	19.03	0.000	0.000	56	279.04	0.000	0.000	78	4091.63	0.000	100.000
13	1.47	0.000	0.000	35	21.50	0.000	0.000	57	315.27	0.000	0.000	79	4622.81	0.000	100.000
14	1.66	0.000	0.000	36	24.29	0.000	0.000	58	356.20	0.000	0.000	80	5222.96	0.000	100.000
15	1.87	0.000	0.000	37	27.45	0.000	0.000	59	402.44	0.000	0.000	81	5901.02	0.000	100.000
16	2.11	0.000	0.000	38	31.01	0.000	0.000	60	454.69	0.000	0.000	82	6667.10	0.000	100.000
17	2.39	0.000	0.000	39	35.03	0.000	0.000	61	513.71	0.000	0.000	83	7532.65	0.000	100.000
18	2.70	0.000	0.000	40	39.58	0.000	0.000	62	580.41	0.000	0.000	84	8510.56	0.000	100.000
19	3.05	0.000	0.000	41	44.72	0.000	0.000	63	655.76	0.000	0.000	85	9615.42	0.000	100.000
20	3.45	0.000	0.000	42	50.53	0.000	0.000	64	740.89	0.000	0.000	86	10863.72	0.000	100.000
21	3.89	0.000	0.000	43	57.09	0.000	0.000	65	837.07	0.000	0.000				
22	4.40	0.000	0.000	44	64.50	0.000	0.000	66	945.74	0.024	0.024				

Explore the future Automotive Test Systems | Process & Environmental | Medical | Semiconductor | Scientific HORIBA

SZ-100

C1M3.nsz

Measurement Results

Date	:	Monday, May 8, 2023 6:39:02 PM
Measurement Type	:	Particle Size
Sample Name	:	C1M3
Scattering Angle	:	90
Temperature of the Holder	:	25.0 °C
Dispersion Medium Viscosity	:	0.895 mPa·s
Transmission Intensity before Meas.	:	22513
Distribution Form	:	Standard
Distribution Form(Dispersity)	:	Monodisperse
Representation of Result	:	Scattering Light Intensity
Count Rate	:	7 kCPS

Calculation Results

Peak No.	S.P.Area Ratio	Mean	S. D.	Mode
1	1.00	928.3 nm	290.2 nm	790.5 nm
2	---	--- nm	--- nm	--- nm
3	---	--- nm	--- nm	--- nm
Total	1.00	928.3 nm	290.2 nm	790.5 nm

Cumulant Operations

Z-Average	:	1313.0 nm
PI	:	0.518

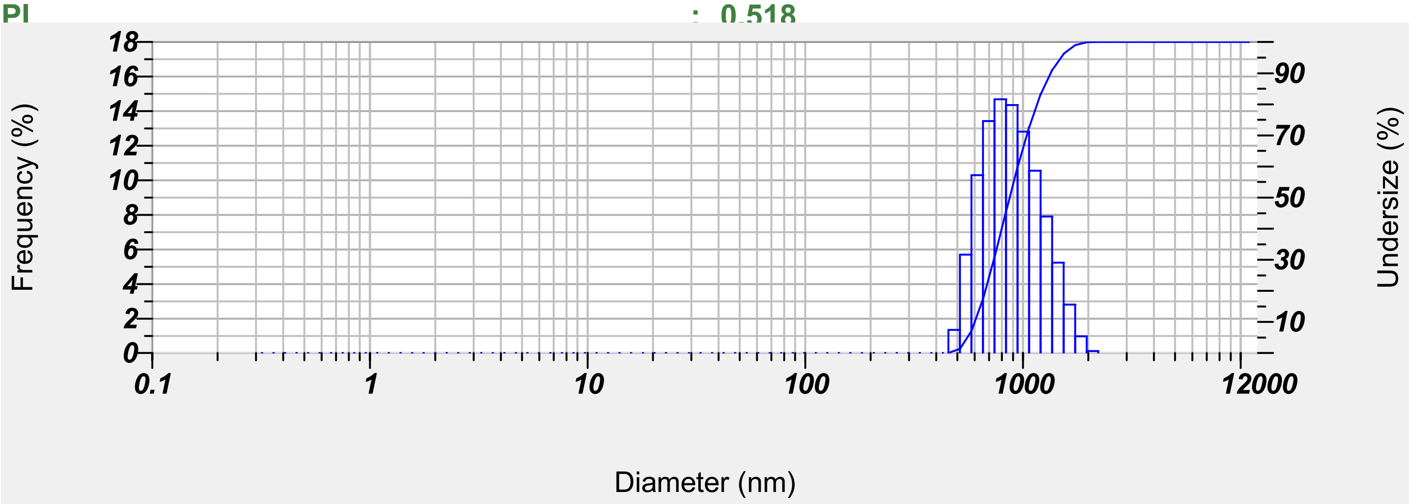

No.	Diameter	Frequency	Cumulation	No.	Diameter	Frequency	Cumulation	No.	Diameter	Frequency	Cumulation	No.	Diameter	Frequency	Cumulation
1	0.34	0.000	0.000	23	4.97	0.000	0.000	45	72.87	0.000	0.000	67	1068.52	12.805	72.501
2	0.38	0.000	0.000	24	5.61	0.000	0.000	46	82.33	0.000	0.000	68	1207.24	10.532	83.033
3	0.43	0.000	0.000	25	6.34	0.000	0.000	47	93.02	0.000	0.000	69	1363.97	7.893	90.927
4	0.49	0.000	0.000	26	7.17	0.000	0.000	48	105.10	0.000	0.000	70	1541.04	5.216	96.142
5	0.55	0.000	0.000	27	8.10	0.000	0.000	49	118.74	0.000	0.000	71	1741.10	2.795	98.937
6	0.62	0.000	0.000	28	9.15	0.000	0.000	50	134.16	0.000	0.000	72	1967.14	0.959	99.896
7	0.70	0.000	0.000	29	10.34	0.000	0.000	51	151.57	0.000	0.000	73	2222.51	0.104	100.000
8	0.80	0.000	0.000	30	11.68	0.000	0.000	52	171.25	0.000	0.000	74	2511.05	0.000	100.000
9	0.90	0.000	0.000	31	13.20	0.000	0.000	53	193.48	0.000	0.000	75	2837.04	0.000	100.000
10	1.02	0.000	0.000	32	14.91	0.000	0.000	54	218.60	0.000	0.000	76	3205.35	0.000	100.000
11	1.15	0.000	0.000	33	16.84	0.000	0.000	55	246.98	0.000	0.000	77	3621.48	0.000	100.000
12	1.30	0.000	0.000	34	19.03	0.000	0.000	56	279.04	0.000	0.000	78	4091.63	0.000	100.000
13	1.47	0.000	0.000	35	21.50	0.000	0.000	57	315.27	0.000	0.000	79	4622.81	0.000	100.000
14	1.66	0.000	0.000	36	24.29	0.000	0.000	58	356.20	0.000	0.000	80	5222.96	0.000	100.000
15	1.87	0.000	0.000	37	27.45	0.000	0.000	59	402.44	0.000	0.000	81	5901.02	0.000	100.000
16	2.11	0.000	0.000	38	31.01	0.000	0.000	60	454.69	0.000	0.000	82	6667.10	0.000	100.000
17	2.39	0.000	0.000	39	35.03	0.000	0.000	61	513.71	1.329	1.329	83	7532.65	0.000	100.000
18	2.70	0.000	0.000	40	39.58	0.000	0.000	62	580.41	5.674	7.004	84	8510.56	0.000	100.000
19	3.05	0.000	0.000	41	44.72	0.000	0.000	63	655.76	10.285	17.288	85	9615.42	0.000	100.000
20	3.45	0.000	0.000	42	50.53	0.000	0.000	64	740.89	13.405	30.693	86	10863.72	0.000	100.000
21	3.89	0.000	0.000	43	57.09	0.000	0.000	65	837.07	14.672	45.366				
22	4.40	0.000	0.000	44	64.50	0.000	0.000	66	945.74	14.330	59.696				

SZ-100

C1M4.nsz

Measurement Results

Date	:	Monday, May 8, 2023 6:36:43 PM
Measurement Type	:	Particle Size
Sample Name	:	C1M4
Scattering Angle	:	90
Temperature of the Holder	:	25.0 °C
Dispersion Medium Viscosity	:	0.896 mPa·s
Transmission Intensity before Meas.	:	24857
Distribution Form	:	Standard
Distribution Form(Dispersity)	:	Monodisperse
Representation of Result	:	Scattering Light Intensity
Count Rate	:	19 kCPS

Calculation Results

Peak No.	S.P.Area Ratio	Mean	S. D.	Mode
1	1.00	1678.4 nm	512.2 nm	1452.8 nm
2	---	--- nm	--- nm	--- nm
3	---	--- nm	--- nm	--- nm
Total	1.00	1678.4 nm	512.2 nm	1452.8 nm

Cumulant Operations

Z-Average	:	1652.5 nm
PI	:	0.714

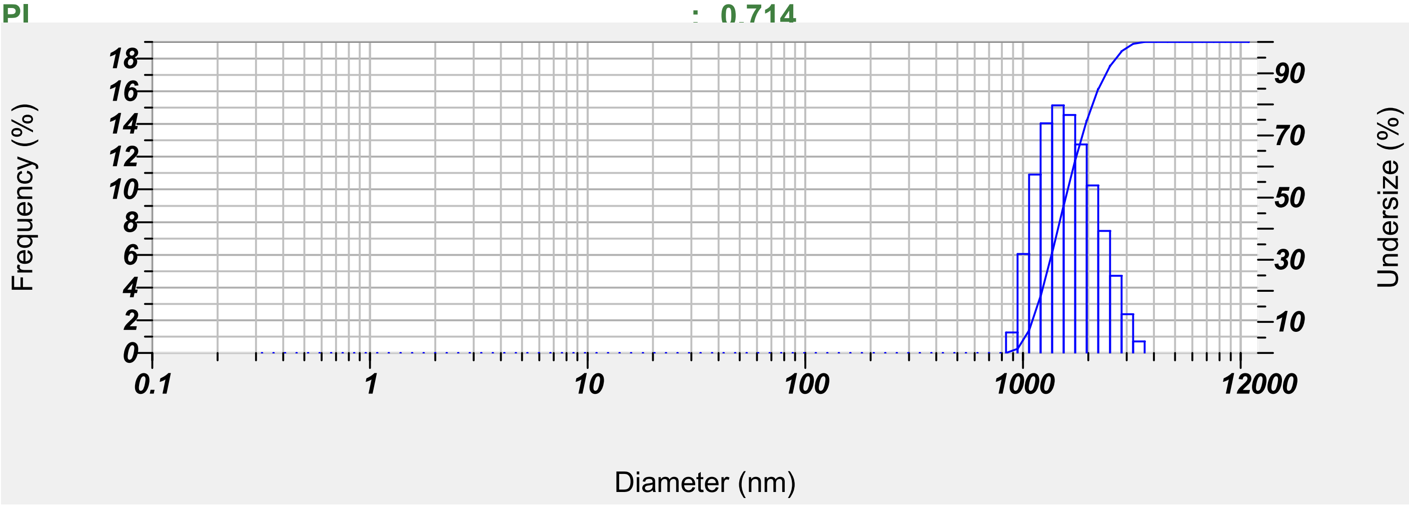

No.	Diameter	Frequency	Cumulation	No.	Diameter	Frequency	Cumulation	No.	Diameter	Frequency	Cumulation	No.	Diameter	Frequency	Cumulation
1	0.34	0.000	0.000	23	4.97	0.000	0.000	45	72.87	0.000	0.000	67	1068.52	6.028	7.275
2	0.38	0.000	0.000	24	5.61	0.000	0.000	46	82.33	0.000	0.000	68	1207.24	10.884	18.160
3	0.43	0.000	0.000	25	6.34	0.000	0.000	47	93.02	0.000	0.000	69	1363.97	14.014	32.174
4	0.49	0.000	0.000	26	7.17	0.000	0.000	48	105.10	0.000	0.000	70	1541.04	15.122	47.296
5	0.55	0.000	0.000	27	8.10	0.000	0.000	49	118.74	0.000	0.000	71	1741.10	14.530	61.826
6	0.62	0.000	0.000	28	9.15	0.000	0.000	50	134.16	0.000	0.000	72	1967.14	12.737	74.563
7	0.70	0.000	0.000	29	10.34	0.000	0.000	51	151.57	0.000	0.000	73	2222.51	10.233	84.796
8	0.80	0.000	0.000	30	11.68	0.000	0.000	52	171.25	0.000	0.000	74	2511.05	7.441	92.237
9	0.90	0.000	0.000	31	13.20	0.000	0.000	53	193.48	0.000	0.000	75	2837.04	4.711	96.948
10	1.02	0.000	0.000	32	14.91	0.000	0.000	54	218.60	0.000	0.000	76	3205.35	2.355	99.303
11	1.15	0.000	0.000	33	16.84	0.000	0.000	55	246.98	0.000	0.000	77	3621.48	0.697	100.000
12	1.30	0.000	0.000	34	19.03	0.000	0.000	56	279.04	0.000	0.000	78	4091.63	0.000	100.000
13	1.47	0.000	0.000	35	21.50	0.000	0.000	57	315.27	0.000	0.000	79	4622.81	0.000	100.000
14	1.66	0.000	0.000	36	24.29	0.000	0.000	58	356.20	0.000	0.000	80	5222.96	0.000	100.000
15	1.87	0.000	0.000	37	27.45	0.000	0.000	59	402.44	0.000	0.000	81	5901.02	0.000	100.000
16	2.11	0.000	0.000	38	31.01	0.000	0.000	60	454.69	0.000	0.000	82	6667.10	0.000	100.000
17	2.39	0.000	0.000	39	35.03	0.000	0.000	61	513.71	0.000	0.000	83	7532.65	0.000	100.000
18	2.70	0.000	0.000	40	39.58	0.000	0.000	62	580.41	0.000	0.000	84	8510.56	0.000	100.000
19	3.05	0.000	0.000	41	44.72	0.000	0.000	63	655.76	0.000	0.000	85	9615.42	0.000	100.000
20	3.45	0.000	0.000	42	50.53	0.000	0.000	64	740.89	0.000	0.000	86	10863.72	0.000	100.000
21	3.89	0.000	0.000	43	57.09	0.000	0.000	65	837.07	0.000	0.000				
22	4.40	0.000	0.000	44	64.50	0.000	0.000	66	945.74	1.247	1.247				

SZ-100

C1M5.nsz

Measurement Results

Date	:	Monday, May 8, 2023 6:31:32 PM
Measurement Type	:	Particle Size
Sample Name	:	C1M5
Scattering Angle	:	90
Temperature of the Holder	:	25.0 °C
Dispersion Medium Viscosity	:	0.896 mPa·s
Transmission Intensity before Meas.	:	24528
Distribution Form	:	Standard
Distribution Form(Dispersity)	:	Monodisperse
Representation of Result	:	Scattering Light Intensity
Count Rate	:	16 kCPS

Calculation Results

Peak No.	S.P.Area Ratio	Mean	S. D.	Mode
1	1.00	1242.5 nm	345.7 nm	1134.0 nm
2	---	--- nm	--- nm	--- nm
3	---	--- nm	--- nm	--- nm
Total	1.00	1242.5 nm	345.7 nm	1134.0 nm

Cumulant Operations

Z-Average	:	1906.8 nm
PI	:	0.998

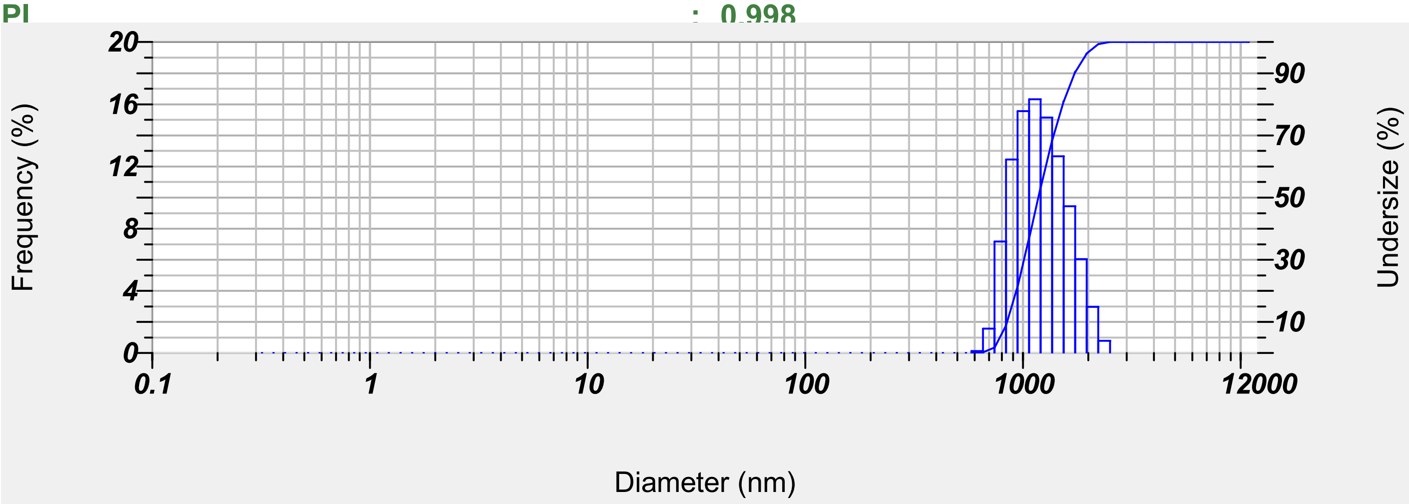

No.	Diameter	Frequency	Cumulation	No.	Diameter	Frequency	Cumulation	No.	Diameter	Frequency	Cumulation	No.	Diameter	Frequency	Cumulation
1	0.34	0.000	0.000	23	4.97	0.000	0.000	45	72.87	0.000	0.000	67	1068.52	15.546	36.792
2	0.38	0.000	0.000	24	5.61	0.000	0.000	46	82.33	0.000	0.000	68	1207.24	16.297	53.089
3	0.43	0.000	0.000	25	6.34	0.000	0.000	47	93.02	0.000	0.000	69	1363.97	15.123	68.213
4	0.49	0.000	0.000	26	7.17	0.000	0.000	48	105.10	0.000	0.000	70	1541.04	12.632	80.844
5	0.55	0.000	0.000	27	8.10	0.000	0.000	49	118.74	0.000	0.000	71	1741.10	9.417	90.261
6	0.62	0.000	0.000	28	9.15	0.000	0.000	50	134.16	0.000	0.000	72	1967.14	6.017	96.278
7	0.70	0.000	0.000	29	10.34	0.000	0.000	51	151.57	0.000	0.000	73	2222.51	2.947	99.225
8	0.80	0.000	0.000	30	11.68	0.000	0.000	52	171.25	0.000	0.000	74	2511.05	0.775	100.000
9	0.90	0.000	0.000	31	13.20	0.000	0.000	53	193.48	0.000	0.000	75	2837.04	0.000	100.000
10	1.02	0.000	0.000	32	14.91	0.000	0.000	54	218.60	0.000	0.000	76	3205.35	0.000	100.000
11	1.15	0.000	0.000	33	16.84	0.000	0.000	55	246.98	0.000	0.000	77	3621.48	0.000	100.000
12	1.30	0.000	0.000	34	19.03	0.000	0.000	56	279.04	0.000	0.000	78	4091.63	0.000	100.000
13	1.47	0.000	0.000	35	21.50	0.000	0.000	57	315.27	0.000	0.000	79	4622.81	0.000	100.000
14	1.66	0.000	0.000	36	24.29	0.000	0.000	58	356.20	0.000	0.000	80	5222.96	0.000	100.000
15	1.87	0.000	0.000	37	27.45	0.000	0.000	59	402.44	0.000	0.000	81	5901.02	0.000	100.000
16	2.11	0.000	0.000	38	31.01	0.000	0.000	60	454.69	0.000	0.000	82	6667.10	0.000	100.000
17	2.39	0.000	0.000	39	35.03	0.000	0.000	61	513.71	0.000	0.000	83	7532.65	0.000	100.000
18	2.70	0.000	0.000	40	39.58	0.000	0.000	62	580.41	0.000	0.000	84	8510.56	0.000	100.000
19	3.05	0.000	0.000	41	44.72	0.000	0.000	63	655.76	0.105	0.105	85	9615.42	0.000	100.000
20	3.45	0.000	0.000	42	50.53	0.000	0.000	64	740.89	1.556	1.662	86	10863.72	0.000	100.000
21	3.89	0.000	0.000	43	57.09	0.000	0.000	65	837.07	7.154	8.815				
22	4.40	0.000	0.000	44	64.50	0.000	0.000	66	945.74	12.432	21.247				

SZ-100

C2M1.nsz

Measurement Results

Date	:	Monday, May 8, 2023 7:08:22 PM
Measurement Type	:	Particle Size
Sample Name	:	C2M1
Scattering Angle	:	90
Temperature of the Holder	:	25.0 °C
Dispersion Medium Viscosity	:	0.895 mPa·s
Transmission Intensity before Meas.	:	24827
Distribution Form	:	Standard
Distribution Form(Dispersity)	:	Monodisperse
Representation of Result	:	Scattering Light Intensity
Count Rate	:	23 kCPS

Calculation Results

Peak No.	S.P.Area Ratio	Mean	S. D.	Mode
1	1.00	992.0 nm	309.1 nm	888.0 nm
2	---	--- nm	--- nm	--- nm
3	---	--- nm	--- nm	--- nm
Total	1.00	992.0 nm	309.1 nm	888.0 nm

Cumulant Operations

Z-Average	:	1288.8 nm
PI	:	0.494

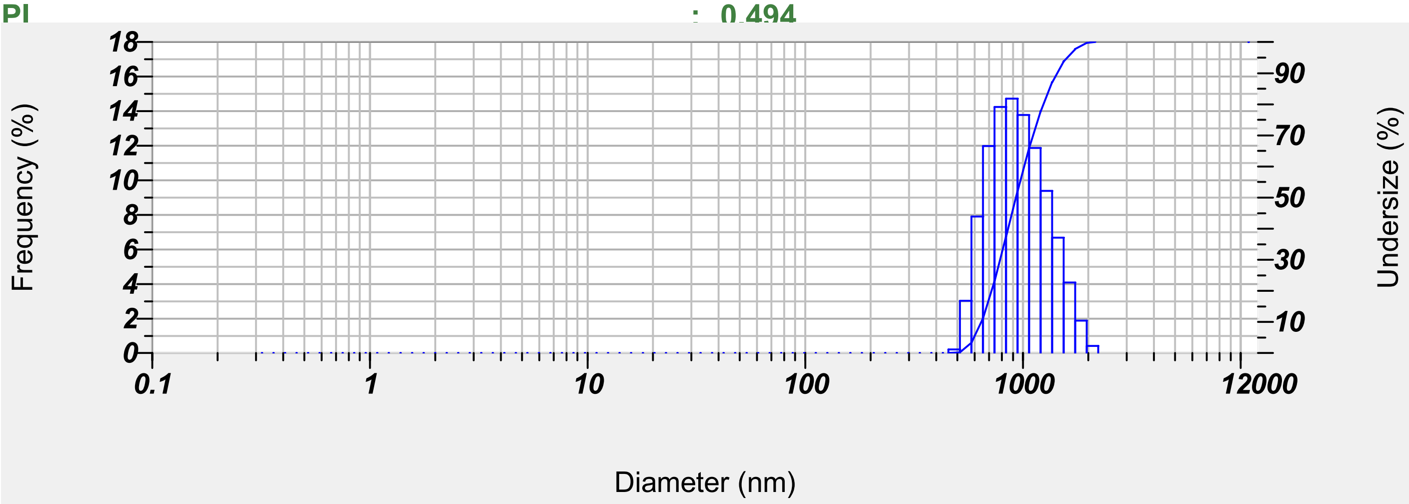

No.	Diameter	Frequency	Cumulation	No.	Diameter	Frequency	Cumulation	No.	Diameter	Frequency	Cumulation	No.	Diameter	Frequency	Cumulation
1	0.34	0.000	0.000	23	4.97	0.000	0.000	45	72.87	0.000	0.000	67	1068.52	13.769	65.772
2	0.38	0.000	0.000	24	5.61	0.000	0.000	46	82.33	0.000	0.000	68	1207.24	11.846	77.618
3	0.43	0.000	0.000	25	6.34	0.000	0.000	47	93.02	0.000	0.000	69	1363.97	9.362	86.980
4	0.49	0.000	0.000	26	7.17	0.000	0.000	48	105.10	0.000	0.000	70	1541.04	6.671	93.651
5	0.55	0.000	0.000	27	8.10	0.000	0.000	49	118.74	0.000	0.000	71	1741.10	4.077	97.728
6	0.62	0.000	0.000	28	9.15	0.000	0.000	50	134.16	0.000	0.000	72	1967.14	1.871	99.598
7	0.70	0.000	0.000	29	10.34	0.000	0.000	51	151.57	0.000	0.000	73	2222.51	0.402	100.000
8	0.80	0.000	0.000	30	11.68	0.000	0.000	52	171.25	0.000	0.000	74	2511.05	0.000	100.000
9	0.90	0.000	0.000	31	13.20	0.000	0.000	53	193.48	0.000	0.000	75	2837.04	0.000	100.000
10	1.02	0.000	0.000	32	14.91	0.000	0.000	54	218.60	0.000	0.000	76	3205.35	0.000	100.000
11	1.15	0.000	0.000	33	16.84	0.000	0.000	55	246.98	0.000	0.000	77	3621.48	0.000	100.000
12	1.30	0.000	0.000	34	19.03	0.000	0.000	56	279.04	0.000	0.000	78	4091.63	0.000	100.000
13	1.47	0.000	0.000	35	21.50	0.000	0.000	57	315.27	0.000	0.000	79	4622.81	0.000	100.000
14	1.66	0.000	0.000	36	24.29	0.000	0.000	58	356.20	0.000	0.000	80	5222.96	0.000	100.000
15	1.87	0.000	0.000	37	27.45	0.000	0.000	59	402.44	0.000	0.000	81	5901.02	0.000	100.000
16	2.11	0.000	0.000	38	31.01	0.000	0.000	60	454.69	0.000	0.000	82	6667.10	0.000	100.000
17	2.39	0.000	0.000	39	35.03	0.000	0.000	61	513.71	0.191	0.191	83	7532.65	0.000	100.000
18	2.70	0.000	0.000	40	39.58	0.000	0.000	62	580.41	3.015	3.206	84	8510.56	0.000	100.000
19	3.05	0.000	0.000	41	44.72	0.000	0.000	63	655.76	7.888	11.093	85	9615.42	0.000	100.000
20	3.45	0.000	0.000	42	50.53	0.000	0.000	64	740.89	11.961	23.054	86	10863.72	0.000	100.000
21	3.89	0.000	0.000	43	57.09	0.000	0.000	65	837.07	14.235	37.289				
22	4.40	0.000	0.000	44	64.50	0.000	0.000	66	945.74	14.714	52.003				

SZ-100

C3M1.nsz

Measurement Results

Date	:	Monday, May 8, 2023 7:20:57 PM
Measurement Type	:	Particle Size
Sample Name	:	C3M1
Scattering Angle	:	90
Temperature of the Holder	:	24.9 °C
Dispersion Medium Viscosity	:	0.897 mPa·s
Transmission Intensity before Meas.	:	25112
Distribution Form	:	Standard
Distribution Form(Dispersity)	:	Monodisperse
Representation of Result	:	Scattering Light Intensity
Count Rate	:	30 kCPS

Calculation Results

Peak No.	S.P.Area Ratio	Mean	S. D.	Mode
1	1.00	929.7 nm	294.8 nm	789.8 nm
2	---	--- nm	--- nm	--- nm
3	---	--- nm	--- nm	--- nm
Total	1.00	929.7 nm	294.8 nm	789.8 nm

Cumulant Operations

Z-Average	:	1144.4 nm
PI	:	0.557

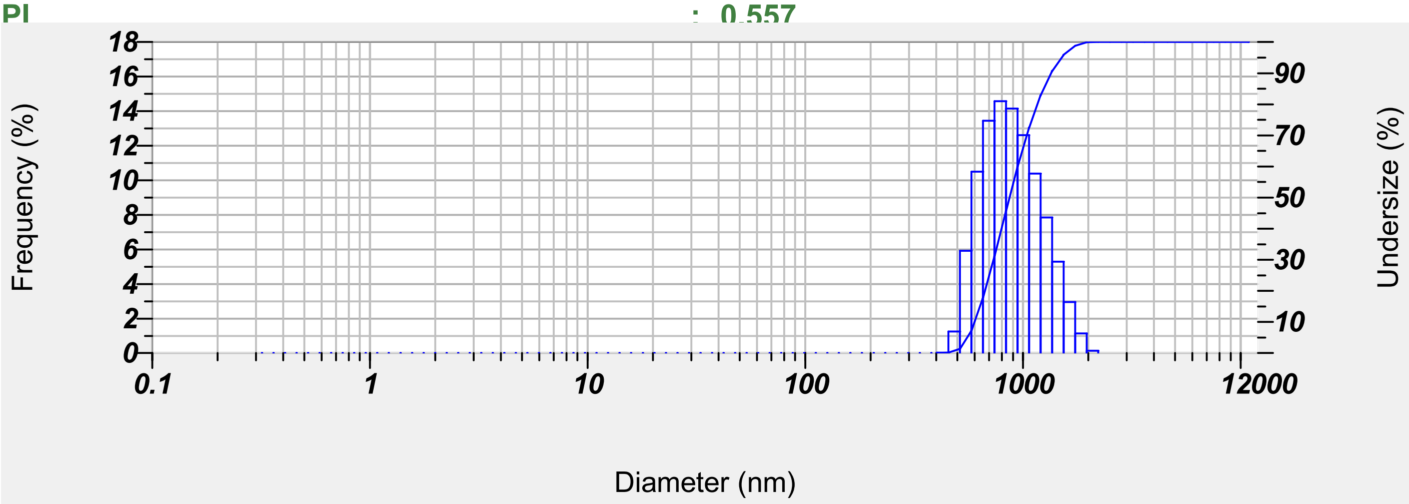

No.	Diameter	Frequency	Cumulation	No.	Diameter	Frequency	Cumulation	No.	Diameter	Frequency	Cumulation	No.	Diameter	Frequency	Cumulation
1	0.34	0.000	0.000	23	4.97	0.000	0.000	45	72.87	0.000	0.000	67	1068.52	12.600	72.339
2	0.38	0.000	0.000	24	5.61	0.000	0.000	46	82.33	0.000	0.000	68	1207.24	10.379	82.719
3	0.43	0.000	0.000	25	6.34	0.000	0.000	47	93.02	0.000	0.000	69	1363.97	7.835	90.554
4	0.49	0.000	0.000	26	7.17	0.000	0.000	48	105.10	0.000	0.000	70	1541.04	5.267	95.821
5	0.55	0.000	0.000	27	8.10	0.000	0.000	49	118.74	0.000	0.000	71	1741.10	2.937	98.758
6	0.62	0.000	0.000	28	9.15	0.000	0.000	50	134.16	0.000	0.000	72	1967.14	1.115	99.873
7	0.70	0.000	0.000	29	10.34	0.000	0.000	51	151.57	0.000	0.000	73	2222.51	0.127	100.000
8	0.80	0.000	0.000	30	11.68	0.000	0.000	52	171.25	0.000	0.000	74	2511.05	0.000	100.000
9	0.90	0.000	0.000	31	13.20	0.000	0.000	53	193.48	0.000	0.000	75	2837.04	0.000	100.000
10	1.02	0.000	0.000	32	14.91	0.000	0.000	54	218.60	0.000	0.000	76	3205.35	0.000	100.000
11	1.15	0.000	0.000	33	16.84	0.000	0.000	55	246.98	0.000	0.000	77	3621.48	0.000	100.000
12	1.30	0.000	0.000	34	19.03	0.000	0.000	56	279.04	0.000	0.000	78	4091.63	0.000	100.000
13	1.47	0.000	0.000	35	21.50	0.000	0.000	57	315.27	0.000	0.000	79	4622.81	0.000	100.000
14	1.66	0.000	0.000	36	24.29	0.000	0.000	58	356.20	0.000	0.000	80	5222.96	0.000	100.000
15	1.87	0.000	0.000	37	27.45	0.000	0.000	59	402.44	0.000	0.000	81	5901.02	0.000	100.000
16	2.11	0.000	0.000	38	31.01	0.000	0.000	60	454.69	0.011	0.011	82	6667.10	0.000	100.000
17	2.39	0.000	0.000	39	35.03	0.000	0.000	61	513.71	1.227	1.238	83	7532.65	0.000	100.000
18	2.70	0.000	0.000	40	39.58	0.000	0.000	62	580.41	5.906	7.144	84	8510.56	0.000	100.000
19	3.05	0.000	0.000	41	44.72	0.000	0.000	63	655.76	10.479	17.623	85	9615.42	0.000	100.000
20	3.45	0.000	0.000	42	50.53	0.000	0.000	64	740.89	13.433	31.056	86	10863.72	0.000	100.000
21	3.89	0.000	0.000	43	57.09	0.000	0.000	65	837.07	14.553	45.608				
22	4.40	0.000	0.000	44	64.50	0.000	0.000	66	945.74	14.131	59.740				

SZ-100

C4M1.nsz

Measurement Results

Date	:	Monday, May 8, 2023 7:29:04 PM
Measurement Type	:	Particle Size
Sample Name	:	C4M1
Scattering Angle	:	90
Temperature of the Holder	:	25.0 °C
Dispersion Medium Viscosity	:	0.895 mPa·s
Transmission Intensity before Meas.	:	25005
Distribution Form	:	Standard
Distribution Form(Dispersity)	:	Monodisperse
Representation of Result	:	Scattering Light Intensity
Count Rate	:	21 kCPS

Calculation Results

Peak No.	S.P.Area Ratio	Mean	S. D.	Mode
1	1.00	956.9 nm	285.4 nm	885.4 nm
2	---	--- nm	--- nm	--- nm
3	---	--- nm	--- nm	--- nm
Total	1.00	956.9 nm	285.4 nm	885.4 nm

Cumulant Operations

Z-Average	:	1049.8 nm
PI	:	0.676

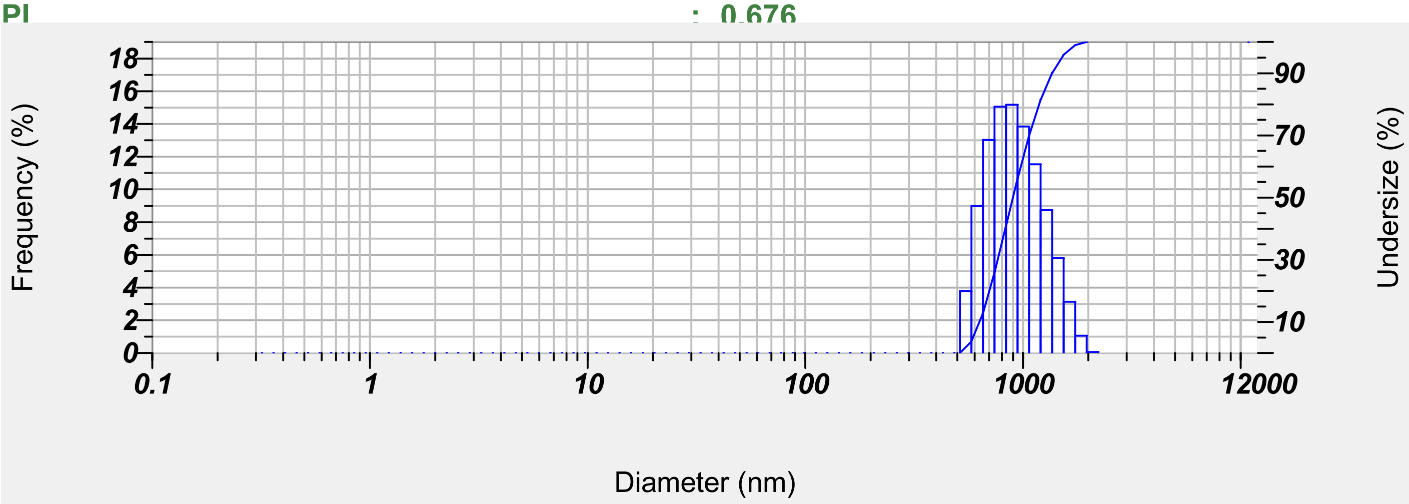

No.	Diameter	Frequency	Cumulation	No.	Diameter	Frequency	Cumulation	No.	Diameter	Frequency	Cumulation	No.	Diameter	Frequency	Cumulation
1	0.34	0.000	0.000	23	4.97	0.000	0.000	45	72.87	0.000	0.000	67	1068.52	13.817	69.765
2	0.38	0.000	0.000	24	5.61	0.000	0.000	46	82.33	0.000	0.000	68	1207.24	11.516	81.281
3	0.43	0.000	0.000	25	6.34	0.000	0.000	47	93.02	0.000	0.000	69	1363.97	8.710	89.991
4	0.49	0.000	0.000	26	7.17	0.000	0.000	48	105.10	0.000	0.000	70	1541.04	5.791	95.781
5	0.55	0.000	0.000	27	8.10	0.000	0.000	49	118.74	0.000	0.000	71	1741.10	3.112	98.893
6	0.62	0.000	0.000	28	9.15	0.000	0.000	50	134.16	0.000	0.000	72	1967.14	1.050	99.944
7	0.70	0.000	0.000	29	10.34	0.000	0.000	51	151.57	0.000	0.000	73	2222.51	0.057	100.000
8	0.80	0.000	0.000	30	11.68	0.000	0.000	52	171.25	0.000	0.000	74	2511.05	0.000	100.000
9	0.90	0.000	0.000	31	13.20	0.000	0.000	53	193.48	0.000	0.000	75	2837.04	0.000	100.000
10	1.02	0.000	0.000	32	14.91	0.000	0.000	54	218.60	0.000	0.000	76	3205.35	0.000	100.000
11	1.15	0.000	0.000	33	16.84	0.000	0.000	55	246.98	0.000	0.000	77	3621.48	0.000	100.000
12	1.30	0.000	0.000	34	19.03	0.000	0.000	56	279.04	0.000	0.000	78	4091.63	0.000	100.000
13	1.47	0.000	0.000	35	21.50	0.000	0.000	57	315.27	0.000	0.000	79	4622.81	0.000	100.000
14	1.66	0.000	0.000	36	24.29	0.000	0.000	58	356.20	0.000	0.000	80	5222.96	0.000	100.000
15	1.87	0.000	0.000	37	27.45	0.000	0.000	59	402.44	0.000	0.000	81	5901.02	0.000	100.000
16	2.11	0.000	0.000	38	31.01	0.000	0.000	60	454.69	0.000	0.000	82	6667.10	0.000	100.000
17	2.39	0.000	0.000	39	35.03	0.000	0.000	61	513.71	0.000	0.000	83	7532.65	0.000	100.000
18	2.70	0.000	0.000	40	39.58	0.000	0.000	62	580.41	3.774	3.774	84	8510.56	0.000	100.000
19	3.05	0.000	0.000	41	44.72	0.000	0.000	63	655.76	8.977	12.751	85	9615.42	0.000	100.000
20	3.45	0.000	0.000	42	50.53	0.000	0.000	64	740.89	13.009	25.760	86	10863.72	0.000	100.000
21	3.89	0.000	0.000	43	57.09	0.000	0.000	65	837.07	15.033	40.792				
22	4.40	0.000	0.000	44	64.50	0.000	0.000	66	945.74	15.155	55.948				

HORIBA Scientific

SZ-100

C100.nsz

Measurement Results

Date	:	Sunday, May 7, 2023 3:25:37 PM
Measurement Type	:	Particle Size
Sample Name	:	C100
Scattering Angle	:	90
Temperature of the Holder	:	25.0 °C
Dispersion Medium Viscosity	:	0.895 mPa·s
Transmission Intensity before Meas.	:	29297
Distribution Form	:	Standard
Distribution Form(Dispersity)	:	Monodisperse
Representation of Result	:	Scattering Light Intensity
Count Rate	:	6 kCPS

Calculation Results

Peak No.	S.P.Area Ratio	Mean	S. D.	Mode
1	1.00	814.2 nm	219.7 nm	782.4 nm
2	---	--- nm	--- nm	--- nm
3	---	--- nm	--- nm	--- nm
Total	1.00	814.2 nm	219.7 nm	782.4 nm

Cumulant Operations

Z-Average	:	816.9 nm
PI	:	0.681

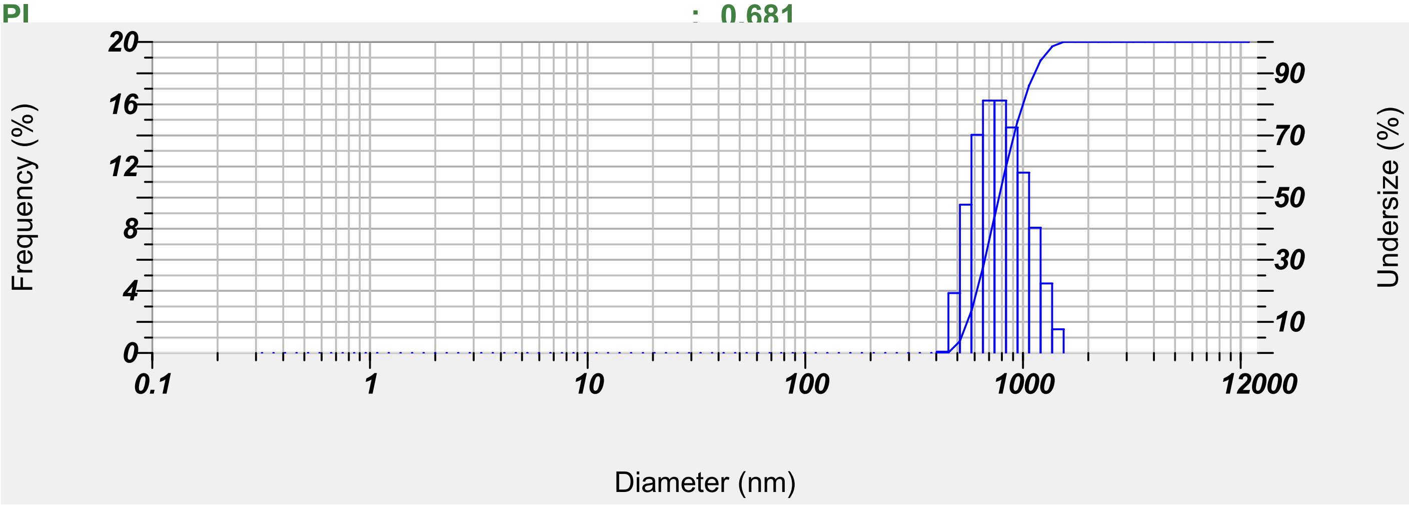

No.	Diameter	Frequency	Cumulation	No.	Diameter	Frequency	Cumulation	No.	Diameter	Frequency	Cumulation	No.	Diameter	Frequency	Cumulation
1	0.34	0.000	0.000	23	4.97	0.000	0.000	45	72.87	0.000	0.000	67	1068.52	11.579	85.979
2	0.38	0.000	0.000	24	5.61	0.000	0.000	46	82.33	0.000	0.000	68	1207.24	8.046	94.025
3	0.43	0.000	0.000	25	6.34	0.000	0.000	47	93.02	0.000	0.000	69	1363.97	4.468	98.493
4	0.49	0.000	0.000	26	7.17	0.000	0.000	48	105.10	0.000	0.000	70	1541.04	1.507	100.000
5	0.55	0.000	0.000	27	8.10	0.000	0.000	49	118.74	0.000	0.000	71	1741.10	0.000	100.000
6	0.62	0.000	0.000	28	9.15	0.000	0.000	50	134.16	0.000	0.000	72	1967.14	0.000	100.000
7	0.70	0.000	0.000	29	10.34	0.000	0.000	51	151.57	0.000	0.000	73	2222.51	0.000	100.000
8	0.80	0.000	0.000	30	11.68	0.000	0.000	52	171.25	0.000	0.000	74	2511.05	0.000	100.000
9	0.90	0.000	0.000	31	13.20	0.000	0.000	53	193.48	0.000	0.000	75	2837.04	0.000	100.000
10	1.02	0.000	0.000	32	14.91	0.000	0.000	54	218.60	0.000	0.000	76	3205.35	0.000	100.000
11	1.15	0.000	0.000	33	16.84	0.000	0.000	55	246.98	0.000	0.000	77	3621.48	0.000	100.000
12	1.30	0.000	0.000	34	19.03	0.000	0.000	56	279.04	0.000	0.000	78	4091.63	0.000	100.000
13	1.47	0.000	0.000	35	21.50	0.000	0.000	57	315.27	0.000	0.000	79	4622.81	0.000	100.000
14	1.66	0.000	0.000	36	24.29	0.000	0.000	58	356.20	0.000	0.000	80	5222.96	0.000	100.000
15	1.87	0.000	0.000	37	27.45	0.000	0.000	59	402.44	0.000	0.000	81	5901.02	0.000	100.000
16	2.11	0.000	0.000	38	31.01	0.000	0.000	60	454.69	0.070	0.070	82	6667.10	0.000	100.000
17	2.39	0.000	0.000	39	35.03	0.000	0.000	61	513.71	3.840	3.910	83	7532.65	0.000	100.000
18	2.70	0.000	0.000	40	39.58	0.000	0.000	62	580.41	9.534	13.444	84	8510.56	0.000	100.000
19	3.05	0.000	0.000	41	44.72	0.000	0.000	63	655.76	14.009	27.453	85	9615.42	0.000	100.000
20	3.45	0.000	0.000	42	50.53	0.000	0.000	64	740.89	16.222	43.676	86	10863.72	0.000	100.000
21	3.89	0.000	0.000	43	57.09	0.000	0.000	65	837.07	16.230	59.906				
22	4.40	0.000	0.000	44	64.50	0.000	0.000	66	945.74	14.494	74.400				

SZ-100

C5M1.nsz

Measurement Results

Date	:	Monday, May 8, 2023 5:45:53 PM
Measurement Type	:	Particle Size
Sample Name	:	C5M1
Scattering Angle	:	173
Temperature of the Holder	:	24.8 °C
Dispersion Medium Viscosity	:	0.898 mPa·s
Transmission Intensity before Meas.	:	17
Distribution Form	:	Standard
Distribution Form(Dispersity)	:	Monodisperse
Representation of Result	:	Scattering Light Intensity
Count Rate	:	9016 kCPS

Calculation Results

Peak No.	S.P.Area Ratio	Mean	S. D.	Mode
1	1.00	165.0 nm	44.4 nm	160.2 nm
2	---	--- nm	--- nm	--- nm
3	---	--- nm	--- nm	--- nm
Total	1.00	165.0 nm	44.4 nm	160.2 nm

Cumulant Operations

Z-Average	:	954.2 nm
PI	:	0.496

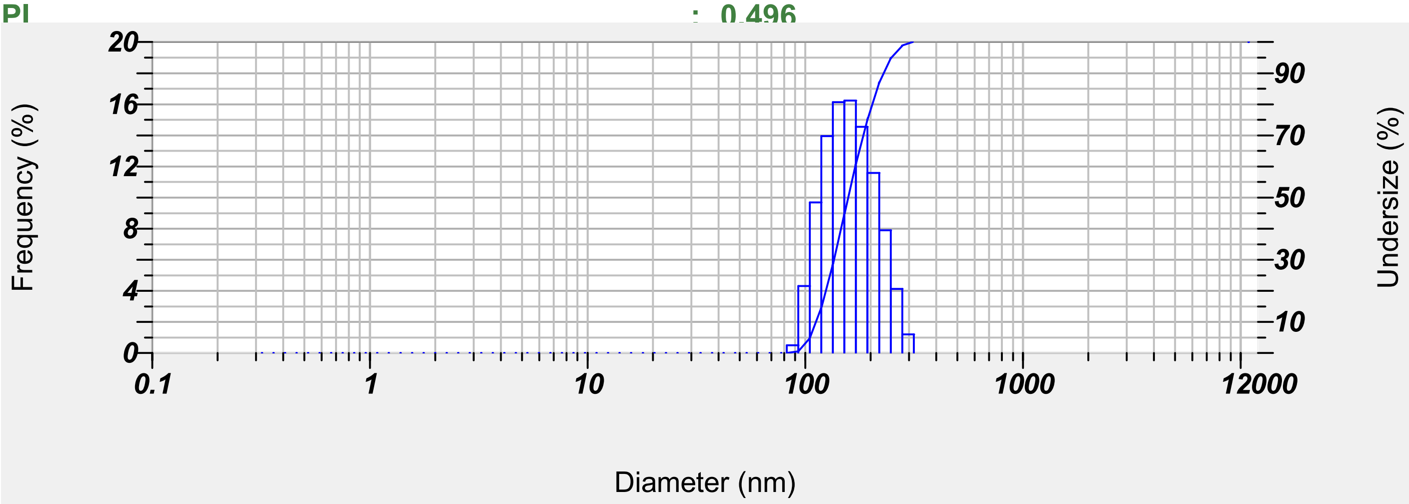

No.	Diameter	Frequency	Cumulation	No.	Diameter	Frequency	Cumulation	No.	Diameter	Frequency	Cumulation	No.	Diameter	Frequency	Cumulation
1	0.34	0.000	0.000	23	4.97	0.000	0.000	45	72.87	0.000	0.000	67	1068.52	0.000	100.000
2	0.38	0.000	0.000	24	5.61	0.000	0.000	46	82.33	0.000	0.000	68	1207.24	0.000	100.000
3	0.43	0.000	0.000	25	6.34	0.000	0.000	47	93.02	0.494	0.494	69	1363.97	0.000	100.000
4	0.49	0.000	0.000	26	7.17	0.000	0.000	48	105.10	4.301	4.795	70	1541.04	0.000	100.000
5	0.55	0.000	0.000	27	8.10	0.000	0.000	49	118.74	9.665	14.460	71	1741.10	0.000	100.000
6	0.62	0.000	0.000	28	9.15	0.000	0.000	50	134.16	13.926	28.386	72	1967.14	0.000	100.000
7	0.70	0.000	0.000	29	10.34	0.000	0.000	51	151.57	16.123	44.509	73	2222.51	0.000	100.000
8	0.80	0.000	0.000	30	11.68	0.000	0.000	52	171.25	16.213	60.722	74	2511.05	0.000	100.000
9	0.90	0.000	0.000	31	13.20	0.000	0.000	53	193.48	14.536	75.258	75	2837.04	0.000	100.000
10	1.02	0.000	0.000	32	14.91	0.000	0.000	54	218.60	11.572	86.830	76	3205.35	0.000	100.000
11	1.15	0.000	0.000	33	16.84	0.000	0.000	55	246.98	7.877	94.707	77	3621.48	0.000	100.000
12	1.30	0.000	0.000	34	19.03	0.000	0.000	56	279.04	4.114	98.821	78	4091.63	0.000	100.000
13	1.47	0.000	0.000	35	21.50	0.000	0.000	57	315.27	1.179	100.000	79	4622.81	0.000	100.000
14	1.66	0.000	0.000	36	24.29	0.000	0.000	58	356.20	0.000	100.000	80	5222.96	0.000	100.000
15	1.87	0.000	0.000	37	27.45	0.000	0.000	59	402.44	0.000	100.000	81	5901.02	0.000	100.000
16	2.11	0.000	0.000	38	31.01	0.000	0.000	60	454.69	0.000	100.000	82	6667.10	0.000	100.000
17	2.39	0.000	0.000	39	35.03	0.000	0.000	61	513.71	0.000	100.000	83	7532.65	0.000	100.000
18	2.70	0.000	0.000	40	39.58	0.000	0.000	62	580.41	0.000	100.000	84	8510.56	0.000	100.000
19	3.05	0.000	0.000	41	44.72	0.000	0.000	63	655.76	0.000	100.000	85	9615.42	0.000	100.000
20	3.45	0.000	0.000	42	50.53	0.000	0.000	64	740.89	0.000	100.000	86	10863.72	0.000	100.000
21	3.89	0.000	0.000	43	57.09	0.000	0.000	65	837.07	0.000	100.000				
22	4.40	0.000	0.000	44	64.50	0.000	0.000	66	945.74	0.000	100.000				

SZ-100

M100.nsz

Measurement Results

Date	:	Sunday, May 7, 2023 3:48:08 PM
Measurement Type	:	Particle Size
Sample Name	:	M100
Scattering Angle	:	90
Temperature of the Holder	:	25.0 °C
Dispersion Medium Viscosity	:	0.896 mPa·s
Transmission Intensity before Meas.	:	30710
Distribution Form	:	Standard
Distribution Form(Dispersity)	:	Monodisperse
Representation of Result	:	Scattering Light Intensity
Count Rate	:	44 kCPS

Calculation Results

Peak No.	S.P.Area Ratio	Mean	S. D.	Mode
1	1.00	603.6 nm	153.3 nm	548.7 nm
2	---	--- nm	--- nm	--- nm
3	---	--- nm	--- nm	--- nm
Total	1.00	603.6 nm	153.3 nm	548.7 nm

Cumulant Operations

Z-Average	:	1658.9 nm
PI	:	0.625

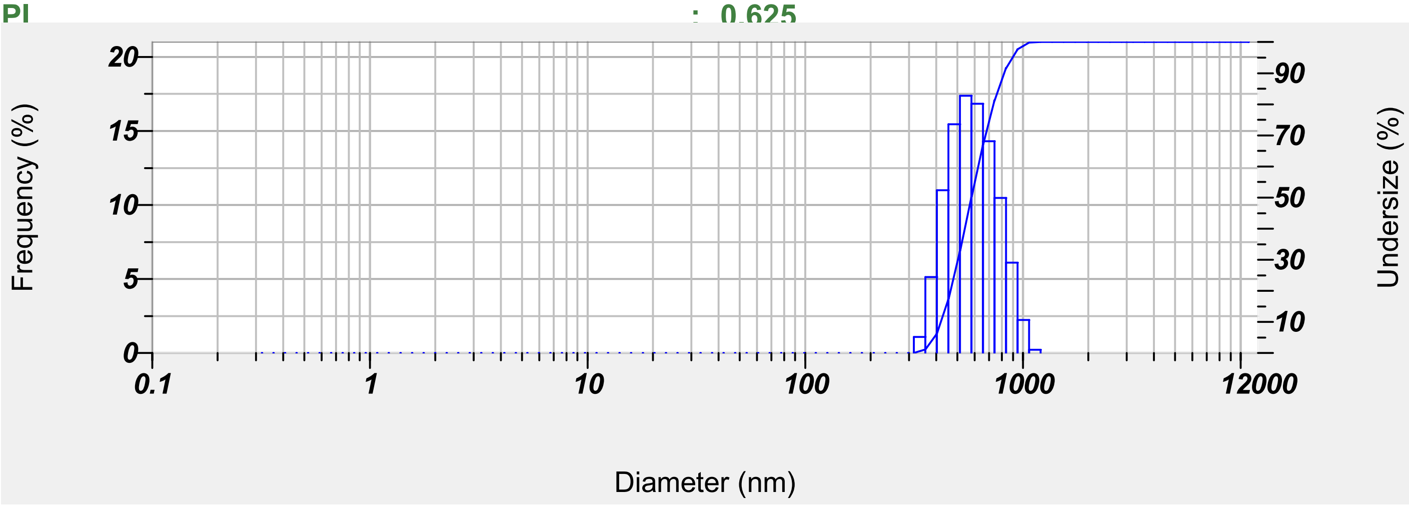

No.	Diameter	Frequency	Cumulation	No.	Diameter	Frequency	Cumulation	No.	Diameter	Frequency	Cumulation	No.	Diameter	Frequency	Cumulation
1	0.34	0.000	0.000	23	4.97	0.000	0.000	45	72.87	0.000	0.000	67	1068.52	2.214	99.794
2	0.38	0.000	0.000	24	5.61	0.000	0.000	46	82.33	0.000	0.000	68	1207.24	0.206	100.000
3	0.43	0.000	0.000	25	6.34	0.000	0.000	47	93.02	0.000	0.000	69	1363.97	0.000	100.000
4	0.49	0.000	0.000	26	7.17	0.000	0.000	48	105.10	0.000	0.000	70	1541.04	0.000	100.000
5	0.55	0.000	0.000	27	8.10	0.000	0.000	49	118.74	0.000	0.000	71	1741.10	0.000	100.000
6	0.62	0.000	0.000	28	9.15	0.000	0.000	50	134.16	0.000	0.000	72	1967.14	0.000	100.000
7	0.70	0.000	0.000	29	10.34	0.000	0.000	51	151.57	0.000	0.000	73	2222.51	0.000	100.000
8	0.80	0.000	0.000	30	11.68	0.000	0.000	52	171.25	0.000	0.000	74	2511.05	0.000	100.000
9	0.90	0.000	0.000	31	13.20	0.000	0.000	53	193.48	0.000	0.000	75	2837.04	0.000	100.000
10	1.02	0.000	0.000	32	14.91	0.000	0.000	54	218.60	0.000	0.000	76	3205.35	0.000	100.000
11	1.15	0.000	0.000	33	16.84	0.000	0.000	55	246.98	0.000	0.000	77	3621.48	0.000	100.000
12	1.30	0.000	0.000	34	19.03	0.000	0.000	56	279.04	0.000	0.000	78	4091.63	0.000	100.000
13	1.47	0.000	0.000	35	21.50	0.000	0.000	57	315.27	0.000	0.000	79	4622.81	0.000	100.000
14	1.66	0.000	0.000	36	24.29	0.000	0.000	58	356.20	1.064	1.064	80	5222.96	0.000	100.000
15	1.87	0.000	0.000	37	27.45	0.000	0.000	59	402.44	5.118	6.182	81	5901.02	0.000	100.000
16	2.11	0.000	0.000	38	31.01	0.000	0.000	60	454.69	10.979	17.161	82	6667.10	0.000	100.000
17	2.39	0.000	0.000	39	35.03	0.000	0.000	61	513.71	15.431	32.592	83	7532.65	0.000	100.000
18	2.70	0.000	0.000	40	39.58	0.000	0.000	62	580.41	17.361	49.954	84	8510.56	0.000	100.000
19	3.05	0.000	0.000	41	44.72	0.000	0.000	63	655.76	16.813	66.766	85	9615.42	0.000	100.000
20	3.45	0.000	0.000	42	50.53	0.000	0.000	64	740.89	14.285	81.051	86	10863.72	0.000	100.000
21	3.89	0.000	0.000	43	57.09	0.000	0.000	65	837.07	10.446	91.497				
22	4.40	0.000	0.000	44	64.50	0.000	0.000	66	945.74	6.083	97.580				

SZ-100

M100.nsz

Measurement Results

Date	:	Sunday, May 7, 2023 3:48:08 PM
Measurement Type	:	Particle Size
Sample Name	:	M100
Scattering Angle	:	90
Temperature of the Holder	:	25.0 °C
Dispersion Medium Viscosity	:	0.896 mPa·s
Transmission Intensity before Meas.	:	30710
Distribution Form	:	Standard
Distribution Form(Dispersity)	:	Monodisperse
Representation of Result	:	Scattering Light Intensity
Count Rate	:	44 kCPS

Calculation Results

Peak No.	S.P.Area Ratio	Mean	S. D.	Mode
1	1.00	603.6 nm	153.3 nm	548.7 nm
2	---	--- nm	--- nm	--- nm
3	---	--- nm	--- nm	--- nm
Total	1.00	603.6 nm	153.3 nm	548.7 nm

Cumulant Operations

Z-Average	:	1658.9 nm
PI	:	0.625

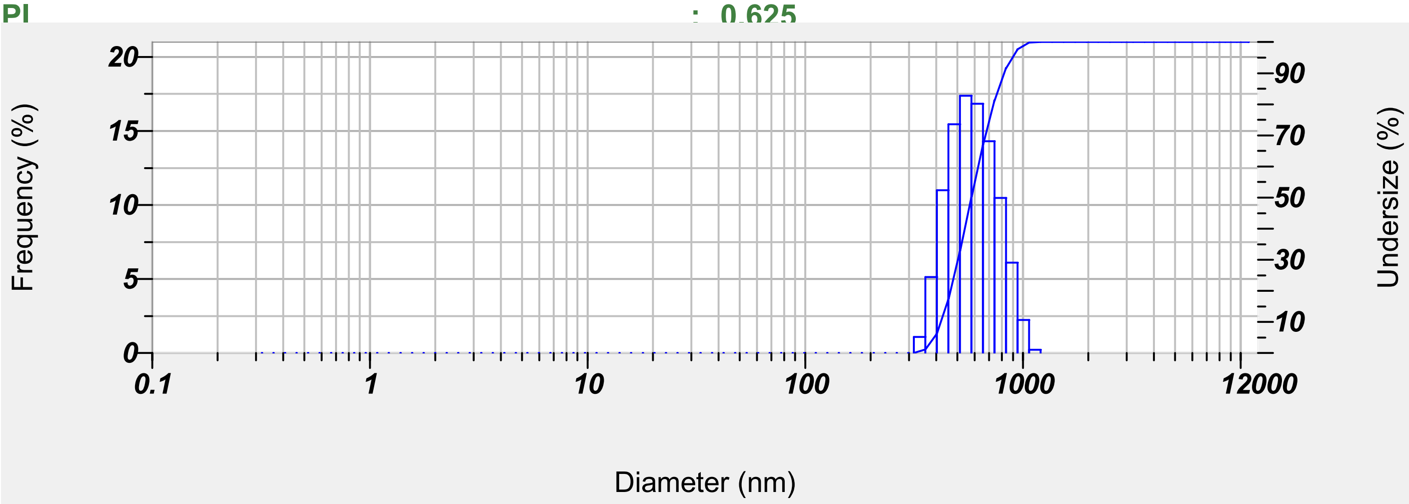

No.	Diameter	Frequency	Cumulation	No.	Diameter	Frequency	Cumulation	No.	Diameter	Frequency	Cumulation	No.	Diameter	Frequency	Cumulation
1	0.34	0.000	0.000	23	4.97	0.000	0.000	45	72.87	0.000	0.000	67	1068.52	2.214	99.794
2	0.38	0.000	0.000	24	5.61	0.000	0.000	46	82.33	0.000	0.000	68	1207.24	0.206	100.000
3	0.43	0.000	0.000	25	6.34	0.000	0.000	47	93.02	0.000	0.000	69	1363.97	0.000	100.000
4	0.49	0.000	0.000	26	7.17	0.000	0.000	48	105.10	0.000	0.000	70	1541.04	0.000	100.000
5	0.55	0.000	0.000	27	8.10	0.000	0.000	49	118.74	0.000	0.000	71	1741.10	0.000	100.000
6	0.62	0.000	0.000	28	9.15	0.000	0.000	50	134.16	0.000	0.000	72	1967.14	0.000	100.000
7	0.70	0.000	0.000	29	10.34	0.000	0.000	51	151.57	0.000	0.000	73	2222.51	0.000	100.000
8	0.80	0.000	0.000	30	11.68	0.000	0.000	52	171.25	0.000	0.000	74	2511.05	0.000	100.000
9	0.90	0.000	0.000	31	13.20	0.000	0.000	53	193.48	0.000	0.000	75	2837.04	0.000	100.000
10	1.02	0.000	0.000	32	14.91	0.000	0.000	54	218.60	0.000	0.000	76	3205.35	0.000	100.000
11	1.15	0.000	0.000	33	16.84	0.000	0.000	55	246.98	0.000	0.000	77	3621.48	0.000	100.000
12	1.30	0.000	0.000	34	19.03	0.000	0.000	56	279.04	0.000	0.000	78	4091.63	0.000	100.000
13	1.47	0.000	0.000	35	21.50	0.000	0.000	57	315.27	0.000	0.000	79	4622.81	0.000	100.000
14	1.66	0.000	0.000	36	24.29	0.000	0.000	58	356.20	1.064	1.064	80	5222.96	0.000	100.000
15	1.87	0.000	0.000	37	27.45	0.000	0.000	59	402.44	5.118	6.182	81	5901.02	0.000	100.000
16	2.11	0.000	0.000	38	31.01	0.000	0.000	60	454.69	10.979	17.161	82	6667.10	0.000	100.000
17	2.39	0.000	0.000	39	35.03	0.000	0.000	61	513.71	15.431	32.592	83	7532.65	0.000	100.000
18	2.70	0.000	0.000	40	39.58	0.000	0.000	62	580.41	17.361	49.954	84	8510.56	0.000	100.000
19	3.05	0.000	0.000	41	44.72	0.000	0.000	63	655.76	16.813	66.766	85	9615.42	0.000	100.000
20	3.45	0.000	0.000	42	50.53	0.000	0.000	64	740.89	14.285	81.051	86	10863.72	0.000	100.000
21	3.89	0.000	0.000	43	57.09	0.000	0.000	65	837.07	10.446	91.497				
22	4.40	0.000	0.000	44	64.50	0.000	0.000	66	945.74	6.083	97.580				
