# Supplementary material for: Chitosan/Carboxymethyl Cellulose Nanocomposites Prepared via Electrolyte Gelation–Spray Drying for Controlled Ampicillin Delivery and Enhanced Antibacterial Activity
Source: Polymers (Basel). 2026 Jan 24;18(3):319. doi: 10.3390/polym18030319 (PMC12899446; doi:10.3390/polym18030319)

ANALYSIS REPORT

Instrument type	
Accessory	
OPUS version	Version 7.5 Build: 7, 5, 18 20140810
Spectrum file name	Ampiciline.0.dpt
Sample name	
Sample form	

Peak pick

Wave number	Abs. intensity	Rel. intensity	Width	Threshold	Shoulder
3444.82	0.162	0.205	424.447	558.09	0
1765.21	0.352	0.166	42.855	79.11	0
1660.23	0.345	0.031	178.244	5.57	0
1599.10	0.308	0.262	160.925	153.79	0
1458.79	0.520	0.015	21.572	11.62	0
1397.75	0.447	0.099	38.188	103.54	0
1323.56	0.482	0.049	38.289	33.63	0
699.74	0.517	0.082	286.166	89.90	0
1129.09	0.589	0.012	21.785	17.08	0
1251.58	0.555	0.014	208.882	8.52	0

Spectrum

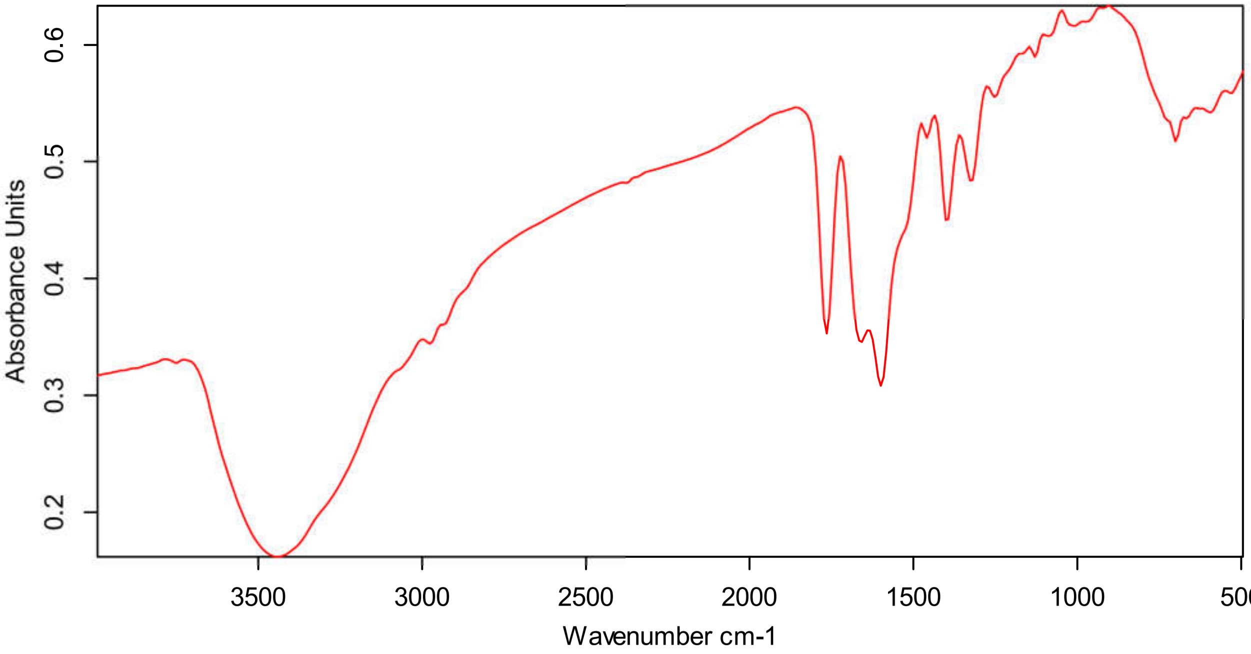

ANALYSIS REPORT

Instrument type	Alpha
Accessory	Sample Compartment *849DC1E2D
OPUS version	Version 7.5 Build: 7, 5, 18 20140810

Spectrum file name	C2M1+AMP.0
Sample name	Sample description
Sample form	Instrument type and / or accessory

Peak pick

Wave number	Abs. intensity	Rel. intensity	Width	Threshold	Shoulder
3443.80	0.111	0.199	446.509	658.25	0
1764.74	0.341	0.069	39.904	35.26	0
1653.36	0.245	0.033	159.989	7.72	0
1596.88	0.239	0.213	173.446	152.75	0
1456.84	0.404	0.008	1741.736	5.29	0
1395.85	0.337	0.091	47.139	101.26	0
1327.04	0.399	0.012	261.650	6.33	0
699.66	0.388	0.067	325.963	66.05	0

Spectrum

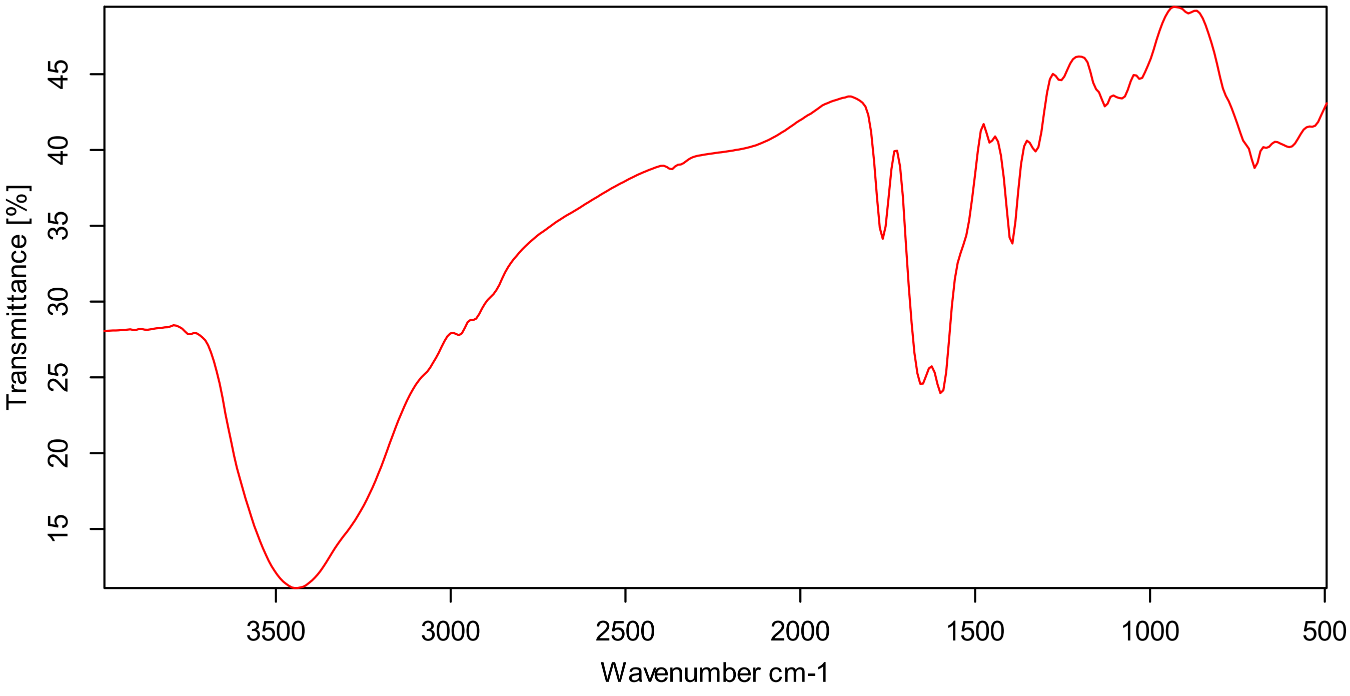

ANALYSIS REPORT

Instrument type	Alpha
Accessory	Sample Compartment *849DC1E2D
OPUS version	Version 7.5 Build: 7, 5, 18 20140810
Spectrum file name	C100.0
Sample name	Sample description
Sample form	Instrument type and / or accessory

Peak pick

Wave number	Abs. intensity	Rel. intensity	Width	Threshold	Shoulder
3449.07	0.425	0.267	378.744	556.88	0
2924.88	0.662	0.021	102.899	37.53	0
1641.57	0.662	0.129	121.173	117.96	0
1383.98	0.733	0.033	111.911	97.99	0
1085.84	0.675	0.100	183.486	226.90	0

Spectrum

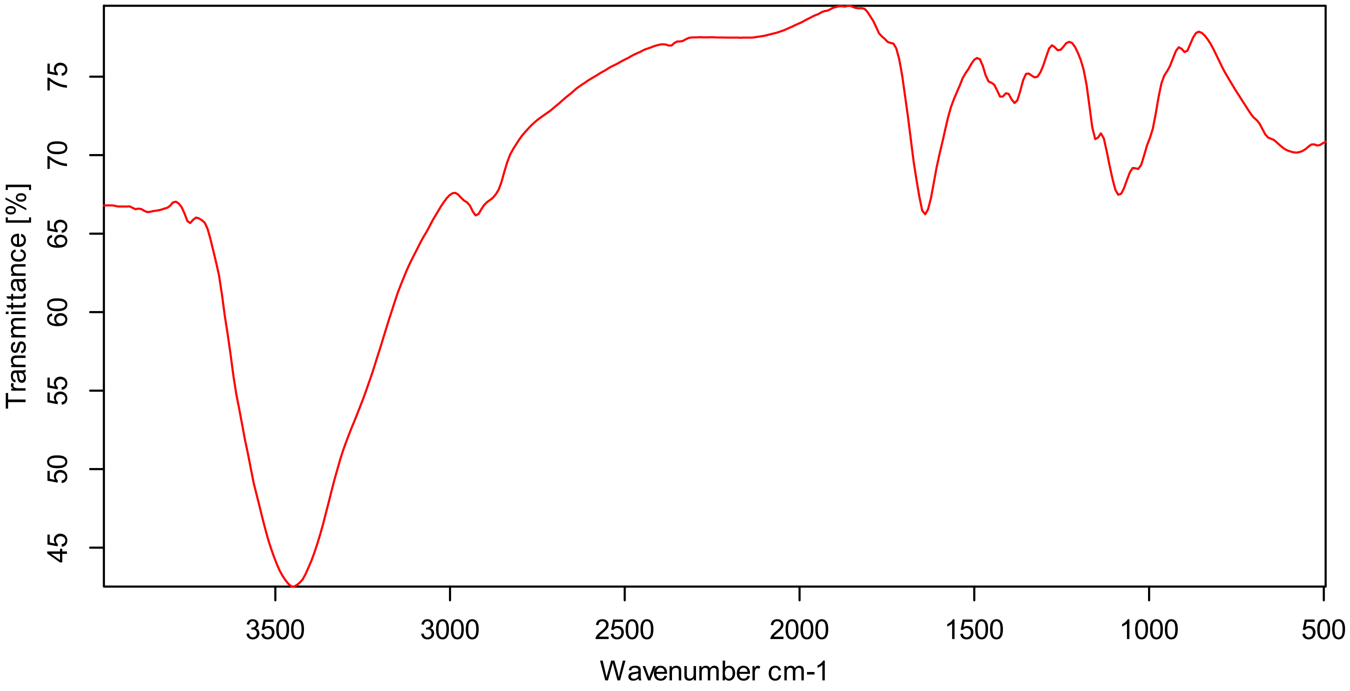

ANALYSIS REPORT

Instrument type	Alpha
Accessory	Sample Compartment *849DC1E2D
OPUS version	Version 7.5 Build: 7, 5, 18 20140810
Spectrum file name	M100.0
Sample name	Sample description
Sample form	Instrument type and / or accessory

Peak pick

Wave number	Abs. intensity	Rel. intensity	Width	Threshold	Shoulder
3450.22	0.281	0.274	394.742	512.56	0
2927.98	0.566	0.009	358.685	12.23	0
1636.45	0.524	0.174	131.927	124.46	0
1420.28	0.629	0.053	69.778	100.62	0
1064.12	0.596	0.121	175.248	85.82	0
1327.93	0.652	0.025	35.517	30.10	0

Spectrum

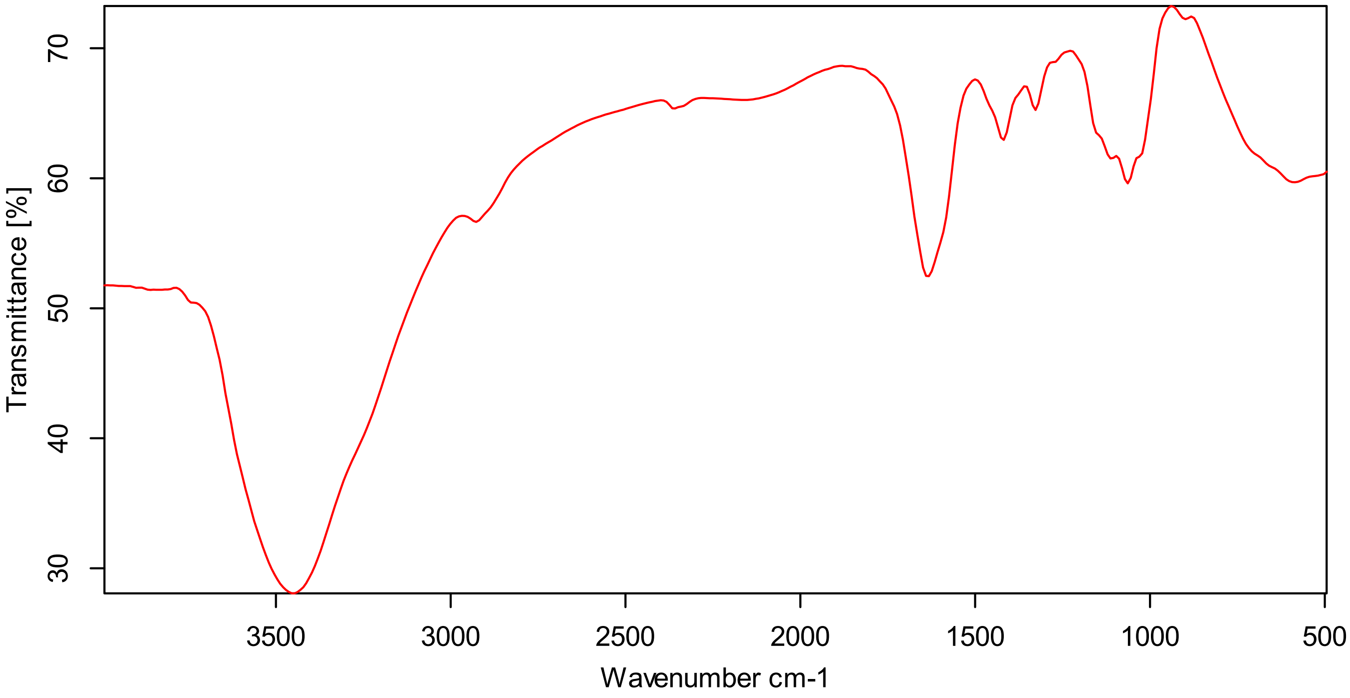

Supplement: Supplementary file 1 [file polymers-18-00319-s001.zip › Figure S3. FTIR of the nanocompoaites and nanocomposite loaded ampicilin.pdf]
